# The Origin and Nature of Tightly Clustered *BTG1* Deletions in Precursor B-Cell Acute Lymphoblastic Leukemia Support a Model of Multiclonal Evolution

**DOI:** 10.1371/journal.pgen.1002533

**Published:** 2012-02-16

**Authors:** Esmé Waanders, Blanca Scheijen, Laurens T. van der Meer, Simon V. van Reijmersdal, Liesbeth van Emst, Yvet Kroeze, Edwin Sonneveld, Peter M. Hoogerbrugge, Ad Geurts van Kessel, Frank N. van Leeuwen, Roland P. Kuiper

**Affiliations:** 1 Department of Human Genetics, Radboud University Nijmegen Medical Centre, Radboud University Centre of Oncology and Nijmegen Centre for Molecular Life Sciences, Nijmegen, The Netherlands; 2 Pediatric Oncology, Radboud University Nijmegen Medical Centre, Radboud University Centre of Oncology and Nijmegen Centre for Molecular Life Sciences, Nijmegen, The Netherlands; 3 Dutch Childhood Oncology Group (DCOG), The Hague, The Netherlands; Cincinnati Children's Hospital Medical Center, United States of America

## Abstract

Recurrent submicroscopic deletions in genes affecting key cellular pathways are a hallmark of pediatric acute lymphoblastic leukemia (ALL). To gain more insight into the mechanism underlying these deletions, we have studied the occurrence and nature of abnormalities in one of these genes, the *B-cell translocation gene 1* (*BTG1*), in a large cohort of pediatric ALL cases. *BTG1* was found to be exclusively affected by genomic deletions, which were detected in 65 out of 722 B-cell precursor ALL (BCP-ALL) patient samples (9%), but not in 109 T-ALL cases. Eight different deletion sizes were identified, which all clustered at the telomeric site in a hotspot region within the second (and last) exon of the *BTG1* gene, resulting in the expression of truncated *BTG1* read-through transcripts. The presence of V(D)J recombination signal sequences at both sites of virtually all deletions strongly suggests illegitimate RAG1/RAG2-mediated recombination as the responsible mechanism. Moreover, high levels of histone H3 lysine 4 trimethylation (H3K4me3), which is known to tether the RAG enzyme complex to DNA, were found within the *BTG1* gene body in BCP-ALL cells, but not T-ALL cells. *BTG1* deletions were rarely found in hyperdiploid BCP-ALLs, but were predominant in other cytogenetic subgroups, including the *ETV6-RUNX1* and *BCR-ABL1* positive BCP-ALL subgroups. Through sensitive PCR-based screening, we identified multiple additional *BTG1* deletions at the subclonal level in BCP-ALL, with equal cytogenetic distribution which, in some cases, grew out into the major clone at relapse. Taken together, our results indicate that *BTG1* deletions may act as “drivers” of leukemogenesis in specific BCP-ALL subgroups, in which they can arise independently in multiple subclones at sites that are prone to aberrant RAG1/RAG2-mediated recombination events. These findings provide further evidence for a complex and multiclonal evolution of ALL.

## Author Summary

Recent studies have alluded to the existence of a complex clonal cellular architecture in acute lymphoblastic leukemia (ALL), where multiple subclones contribute to leukemogenesis. Here, we show that in pediatric B-cell precursor ALL (BCP-ALL) monoallelic deletions in the tumor suppressor *BTG1* locus, which were found to occur in 9% of the patients studied, result in truncations of the gene rather than in complete allelic losses. Using both genetic and epigenetic approaches, we show that these deletions most likely originate from illegitimate RAG recombination. Sensitive backtracking using deletion-spanning PCRs revealed that these *BTG1* deletions occur in specific BCP-ALL subtypes, with frequencies higher than previously anticipated, often in one minor subclone or in multiple independent subclones within individual patients. Subclones that carry a *BTG1* deletion at diagnosis can evolve into the major clone at relapse. These findings link a mechanism of tumor suppressor gene deletion to the multiclonal evolution of ALL.

## Introduction

Acute lymphoblastic leukemia (ALL) is the most common form of cancer in children, and can be subdivided in T-lineage leukemia (T-ALL) and B-cell precursor leukemia (BCP-ALL). BCP-ALL makes up about 80% of all pediatric ALL cases, and comprises genetically distinct subtypes. These ALL subtypes are characterized by specific genetic abnormalities, including aneuploidies (hyperdiploidy and hypodiploidy) and chromosomal translocations leading to *ETV6-RUNX1*, *E2A*-*PBX1*, *BCR-ABL1*, and *MLL* gene fusions [1]. An additional layer of complexity was recently revealed by the identification of recurrent copy number alterations (CNAs), including single gene deletions [2]–[4], some of which are strongly associated with disease progression and outcome [5]–[7]. Despite the high number of CNAs that has been identified in BCP-ALL, the total number of structural genetic lesions in each individual leukemia sample is usually limited to less than 10 per case. A subset of these genetic lesions, predominantly single gene deletions, may act as ‘drivers’ in the development of BCP-ALL. Genes commonly affected by these driver deletions function in key pathways such as cell cycle regulation (*CDKN2A*, *CDKN2B*, *RB1*), lymphoid development (*PAX5*, *EBF1*, *IKZF1*, *RAG1/2*), and nuclear hormone receptor signaling (*NR3C1*, *TBL1XR1*).

One of the newly identified players in BCP-ALL is the *B cell translocation gene-1* (*BTG1*) [4], [8]. *BTG1* belongs to the *BTG/TOB* family of anti-proliferative genes (*BTG1*-*BTG4*, *TOB1* and *TOB2*) and expression of their gene products is altered in a variety of different cancers [9]. The *BTG1* gene has been reported to be affected by deletions in approximately 10% of the BCP-ALL cases [2], [3], and to be targeted by nonsense and missense point mutations in diffuse large B-cell lymphoma [10]. BTG1 was shown to directly interact with multiple transcription factors and modulators involved in the regulation of crucial cellular processes including mRNA turnover and histone modification [11]–[14]. Furthermore, we recently demonstrated that BTG1 regulates the glucocorticoid receptor (GR)-dependent transcriptional response in leukemic cells [8], which turns this gene into an interesting target for modulating therapy response.

Several recurrent chromosomal abnormalities in leukemia show a clustering of their breakpoints, suggesting that features intrinsic to the DNA may underlie their origin. A common mechanism underlying these abnormalities is non-homologous end joining (NHEJ). NHEJ is employed by human cells to repair DNA double strand breaks caused by external damage or by intermediate steps of V(D)J recombination and class switch recombination (CSR) events during lymphocyte development [15]–[17]. The RAG complex mediates V(D)J recombination through the recognition of recombination signal sequences (RSS) located directly adjacent to the V, D, or J segments, thereby generating the variability of immunoglobulins (Ig) as well as B- and T-cell receptors. Illegitimate RAG-mediated recombination has been proposed to be involved in the translocation process of the *LMO2* and *TAL1* proto-oncogenes with *TCRδ* [18], and appears to be the cause of recurrent intragenic deletions of the *IKZF1* gene in BCP-ALL and lymphoid blast crisis in chronic myeloid leukemia (CML) [19], [20].

Comprehensive genomic analyses of relapsed BCP-ALL have revealed that diagnosis and relapse samples are clonally related, but may exhibit subtle differences, frequently involving lesions detected at the time of diagnosis that are absent at the time of relapse [5], [21], [22]. Furthermore, diagnosis and relapse samples may carry alternative lesions affecting genes like e.g. *CDKN2A* and *PAX5*, suggesting that these lesions occur repeatedly during clonal evolution of BCP-ALL [5]. These findings are in line with the generally accepted concept that each leukemic outgrowth comprises a heterogeneous population of leukemic cells of which only a subset has the potential to drive progression of the disease, survive therapy, and/or induce relapse development [23]. Recent studies in BCP-ALL have revealed that this multiclonal architecture supports a complex model of multiclonal evolution [24], [25]. During disease progression and after post-treatment relapse, shifts can occur in the dominance of leukemic subclones, most likely triggered by new selective bottlenecks, whereas the subclonal diversity appears to be maintained.

In the present study, we have characterized the origin and nature of *BTG1* deletions in a cohort of 831 pediatric ALL cases. We demonstrate that *BTG1* deletions are exclusively found in BCP-ALL, both in the predominant clone and in (multiple) minor subclones, and are strongly associated with *ETV6-RUNX1* and *BCR*-*ABL1* positive ALL, but rare in hyper-diploid cases. Furthermore, we show that these deletions most likely arise from illegitimate RAG-mediated recombination, are tightly clustered, and virtually all share their telomeric breakpoint within the second exon of *BTG1*, resulting in the expression of a read-through transcript that gives rise to highly instable truncated BTG1 protein. These results provide important insight into the mechanism underlying the occurrence of *BTG1* deletions in BCP-ALL and support the recently observed complexity of multiclonal evolution of ALL.

## Results

### *BTG1* Is Targeted by Deletions in Specific Subsets of BCP-ALL

We screened 831 pediatric ALL diagnosis samples for copy number aberrations in *BTG1* (Entrez Gene: 694) using multiplex ligation-dependent probe amplification (MLPA). In 722 BCP-ALL samples, we found a total of 65, predominantly monoallelic, deletions (9%), whereas no *BTG1* copy number loss was detected in any of the T-ALL samples (n = 109; P = 0.001; Table 1). In addition, MLPA performed on genomic DNA derived from BCP-ALL (n = 10) and T-ALL (n = 5) cell lines revealed *BTG1* deletions in the BCP-ALL cell lines REH, SUP-B15, MUTZ5 and 380, while none could be detected in the T-ALL cell lines (see Materials and Methods). Sequencing of the entire gene in 135 BCP-ALL cases and of exon 2, which contains the major body of the open reading frame, in 158 additional BCP-ALL cases and 44 T-ALL cases revealed no mutations. Furthermore, bisulfite sequence analysis showed absence of *BTG1* promoter hypermethylation in 20 BCP-ALL and 5 T-ALL cases.

**Table 1:** *BTG1* microdeletion occurrence within cytogenetic subgroups of ALL.

	Number of Samples^a^	*BTG1* deletion positive cases (%)	*BTG1* deletion negative cases (%)	P-value Chi-square
T-ALL	109	0 (0)	109 (100)	0.001
BCP-ALL	722	65 (9.0)	657 (91.0)	
Cytogenetic subgroup				<0.001
*Hyperdiploid (>50chr)*				0.002
Yes	160	5 (3.1)	155 (96.9)	
No	477	55 (11.5)	422 (88.5)	
*ETV6-RUNX1*				<0.001
Yes	142	27 (19.0)	115 (81.0)	
No	371	22 (5.9)	349 (94.1)	
*BCR-ABL1*				0.003
Yes	23	6 (26.1)	17 (73.9)	
No	625	52 (8.3)	573 (91.7)	
*MLL rearranged*				0.385^d^
Yes	17	0 (0)	17 (100)	
No	632	55 (8.7)	577 (91.3)	
*Other subgroup* b				0.155
Yes	180	13 (7.2)	167 (92.8)	
No	342	38 (11.1)	304 (88.9)	
*Subgroup unknown* c				0.245
Yes	200	14 (7.0)	186 (93.0)	
No	522	51 (9.8)	471 (90.2)	

*BTG1* deletion status was determined using MLPA.

a Because of missing values, numbers do not always add up to 722 BCP-ALL cases. Data was available for 637 cases on hyperdiploidy; 513 cases for *ETV6-RUNX1*; 648 cases for *BCR-ABL1;* 649 cases for *MLL*-rearranged.

b The ‘other’ subgroup encompasses cases negative for *ETV6-RUNX1* or *BCR-ABL1* translocations, *MLL*-rearrangement and/or hyperdiploidy. This group includes 10 cases with *E2A-PBX1* translocation, of which none harbor a *BTG1* deletion.

c Subgroup unknown includes all cases in which no data is available in one or more cytogenetic classifications.

d Fisher's exact test was used when sample groups were small.

*BTG1* deletions were found to be unevenly distributed between the different cytogenetic subgroups, being enriched in *ETV6*-*RUNX1* (*TEL*-*AML1*) and *BCR*-*ABL1* positive cases, and less frequent in hyperdiploid cases (P<0.001, P = 0.003 and P = 0.002, respectively; Table 1). In addition, targeted copy number analysis of recurrently affected genes in ALL revealed that cases with *BTG1* deletions more frequently harbored deletions of *ETV6*, *RB1* and *EBF1* (P = 0.007, P<0.001 and P<0.001 respectively; Table S1). Together, our data indicate that *BTG1* is affected by deletions in specific subsets of pediatric BCP-ALL patients.

### A Deletion Breakpoint Hotspot Maps within the Second Exon of *BTG1*

**Figure 1:**
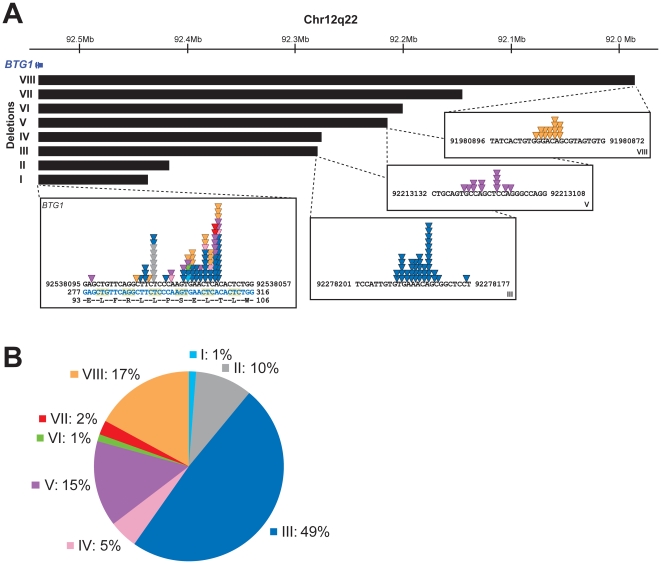
*BTG1* deletions cluster tightly and disrupt the *BTG1* open reading frame. (A) The *BTG1* gene located on the antisense strand of chromosome 12q22 is exclusively disrupted by deletions (indicated by black bars). We identified 8 different deletions ranging from 101 to 557 kb in size. The deletion breakpoints cluster tightly within exon 2, the majority of which are located within a stretch of 10 base pairs. Colored triangles indicate the position of the breakpoints in different patients. Light blue represents the breakpoint of deletion I, grey deletion II, blue deletion III, pink deletion IV, purple deletion V, green deletion VI, red deletion VII and orange deletion VIII, respectively. The exact *BTG1* deletion breakpoint sequences are listed in Table S2. Chromosomal location refers to human GRCh37/hg19 genome assembly. (B) In our cohort of 65 deletion positive BCP-ALL cases, as determined by MLPA, we detected eight distinct deletions with different frequencies. Deletion III was most prevalent (49% of the cases).

To further define the size and location of the *BTG1* deletions, SNP-based genomic profiling was performed on 24 *BTG1* deletion-positive BCP-ALL diagnosis samples and the BCP-ALL derived cell lines REH, SUP-B15, and 380. In nearly all cases, the telomeric deletion breakpoint was located within the *BTG1* gene. Subsequent PCR-based fine-mapping and direct sequencing revealed that the intragenic breakpoints tightly clustered within a region of 33 base pairs in the second (and last) exon of *BTG1*, with the majority (75%) of the breakpoints being located within a stretch of 10 base pairs (Figure 1A, Table S2). The centromeric breakpoints mapped to 8 different positions downstream of the *BTG1* gene, resulting in deletions ranging in size from 101 to 557 kb (deletions I-VIII). This highly specific clustering of deletion breakpoints allowed us to perform a PCR-based screening of all 65 BCP-ALL diagnosis samples. Hence, we were able to exactly map the deletions in 52 of the 65 cases and found that deletion III was most prevalent, comprising almost half of all lesions identified (49%). Deletions V and VIII were found in 15 and 17% of the cases, respectively (Figure 1B). These findings illustrate the high degree of clustering of *BTG1* deletion breakpoints in BCP-ALL.

### Subclones with Distinct *BTG1* Deletions Frequently Arise in BCP-ALL Subtypes

**Figure 2:**
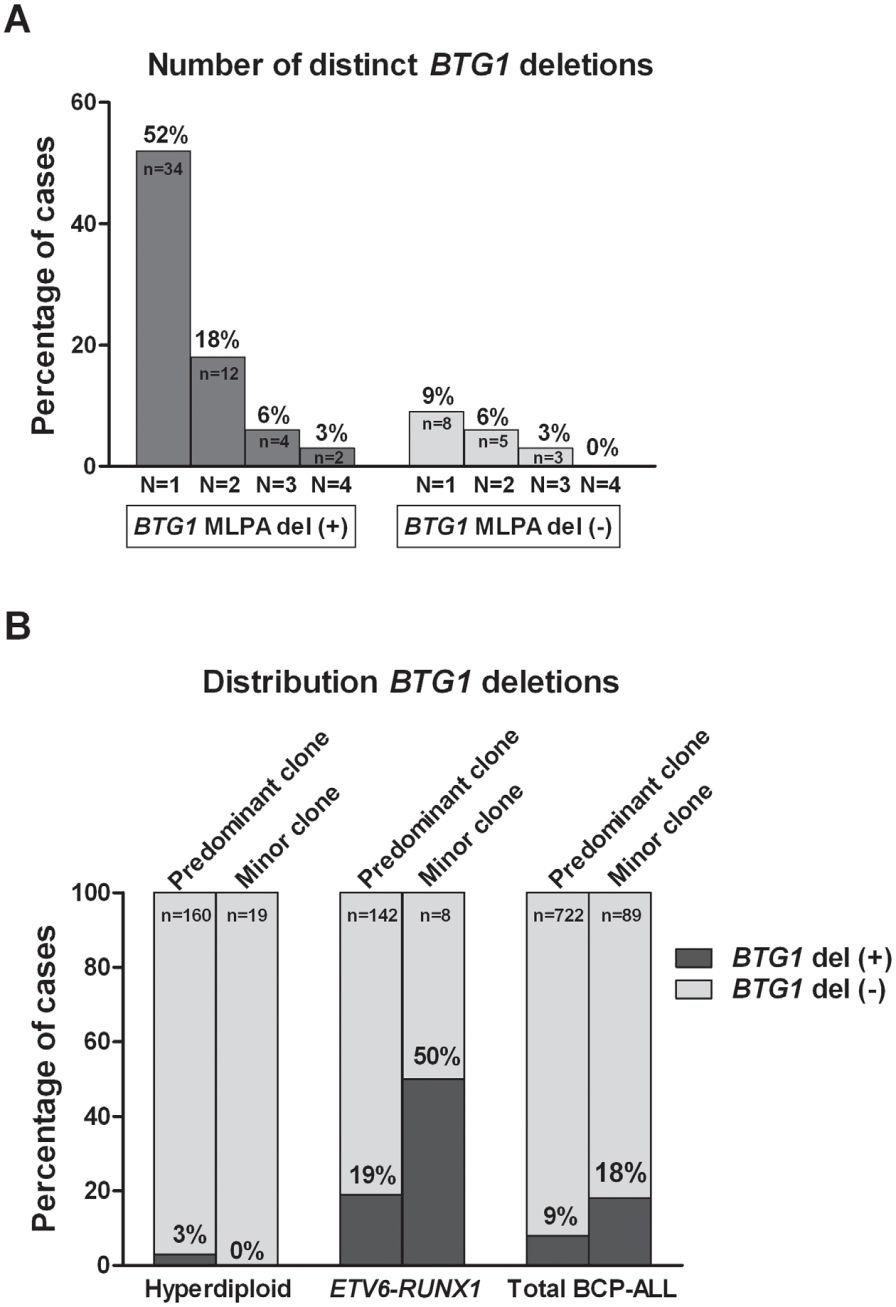
Multiple *BTG1* deletion-positive clones are present in specific BCP-ALL subtypes. (A) Recurrence of multiclonal *BTG1* deletions. A sensitive PCR method was used to screen for eight different deletion breakpoints (deletion I–VIII) in *BTG1* MLPA deletion positive (+) cases (n = 65), and to screen for the three most frequent deletion breakpoints (deletion III, V and VIII) in *BTG1* MLPA deletion negative (−) cases (n = 89). (B) *BTG1* deletion frequency in the two major cytogenetic subgroups of BCP-ALL (Hyperdiploid and *ETV6*-*RUNX1*). Presence of a *BTG1* deletion in the predominant clone was determined by MLPA on the entire cohort of BCP-ALL cases (n = 722), and was compared to deletions detected as a minor clone in MLPA-negative cases (n = 89) by deletion-spanning PCR. Distributions are similar, being depleted from hyperdiploid cases and enriched in *ETV6*-*RUNX1*-positive cases as compared to the total group.

Remarkably, while validating the *BTG1* deletions with breakpoint-spanning PCRs, multiple *BTG1* deletions were identified in 18 of the 65 deletion positive cases (27%), all of which turned out to be monoallelic losses using MLPA. The individual deletions appeared to be unique in their exact breakpoints and flanking interstitial sequences (Table S2), thus indicating the presence of multiple subclones which independently acquired *BTG1* deletions. In one patient, as many as 4 unique deletions were identified (Figure 2A, Table S3). Triggered by this finding we performed a similar PCR-based *BTG1* deletion screening for the three most common deletions (III, V, and VIII) in 89 deletion negative BCP-ALL cases as scored by MLPA or SNP array and were able to confirm the presence *BTG1* deletions in minor subclones in 16 cases (18%), which is substantially more frequent than the occurrence of this abnormality in the predominant leukemic clone (Figure 2B). Similar to the previously observed clonal *BTG1* deletions, these subclonal events were detected in several *ETV6-RUNX1*–positive cases and not in the hyperdiploid subgroup (Figure 2B, Table S4). Furthermore, no subclonal *BTG1* deletions were detected in 77 T-ALL cases nor in 26 bone marrow samples from healthy volunteers. Together, we conclude that *BTG1* deletions are associated with specific BCP-ALL subtypes where they can repeatedly arise in independent subclones.

### *BTG1* Deletion (Sub)Clones May Reoccur in Relapse

To examine whether minor subclones or progenitors thereof that harbor a *BTG1* deletion can evolve into relapse clones, we analyzed 62 matched relapse samples using MLPA for focal *BTG1* deletions. All deletion-positive cases were subjected to breakpoint-spanning PCR for each of the eight deletion variants (deletions I–VIII). Again, we found unique *BTG1* deletions both in the predominant clone and/or in (multiple) subclones (Table 2). An additional deletion was sequenced of which the telomeric breakpoint is located 2 kb downstream in the 3′UTR of *BTG1*. Backtracking and sequencing of the deletion breakpoints in matched diagnosis samples revealed that these *BTG1* deletion clones originated from a (sub)clone at diagnosis in at least four cases. These data confirm that *BTG1* deletions arise repeatedly and independently during disease progression and that these leukemic (sub)clones frequently reoccur at relapse.

**Table 2:** *BTG1* deletions in relapsed cases.

Case	Relapse (MLPA)	Relapse (PCR)	Diagnosis (MLPA)	Diagnosis (PCR)	Backtracking of relapse clone in diagnosis sample^a^
BCP-ALL 1078	deletion	III, IV	normal	III	two new events
BCP-ALL 1174	deletion	III	normal	III	match diagnosis and relapse
BCP-ALL 1193	deletion	IV	normal	N.D.^b^	one new event
BCP-ALL 1236	deletion	N.D.^b^	normal	N.D.^b^	-
BCP-ALL 1405	deletion	III, VIII	normal	III, VIII	two new events
BCP-ALL 1086	deletion	VI	deletion	VI	match diagnosis and relapse
BCP-ALL 1176	deletion	N.D.^b^	deletion	N.D.^b^	-
BCP-ALL 1304	deletion	III, VIIIb^c^	deletion	VIIIb^c^	new event + match diagnosis and relapse
BCP-ALL 1273	deletion^d^	N.D.^b^	deletion	III	new event

a Determined by sequence analysis of breakpoint spanning PCR product.

b The deletion breakpoint could not be detected (N.D.) using the eight breakpoint-spanning PCR assays (I–VIII).

c The breakpoint does not cluster within *BTG1* exon 2, but is located 2 kb downstream in the 3′UTR.

d Homozygous deletion.

### Truncated *BTG1* Transcripts Are Expressed in BCP-ALLs with *BTG1* Deletions

**Figure 3:**
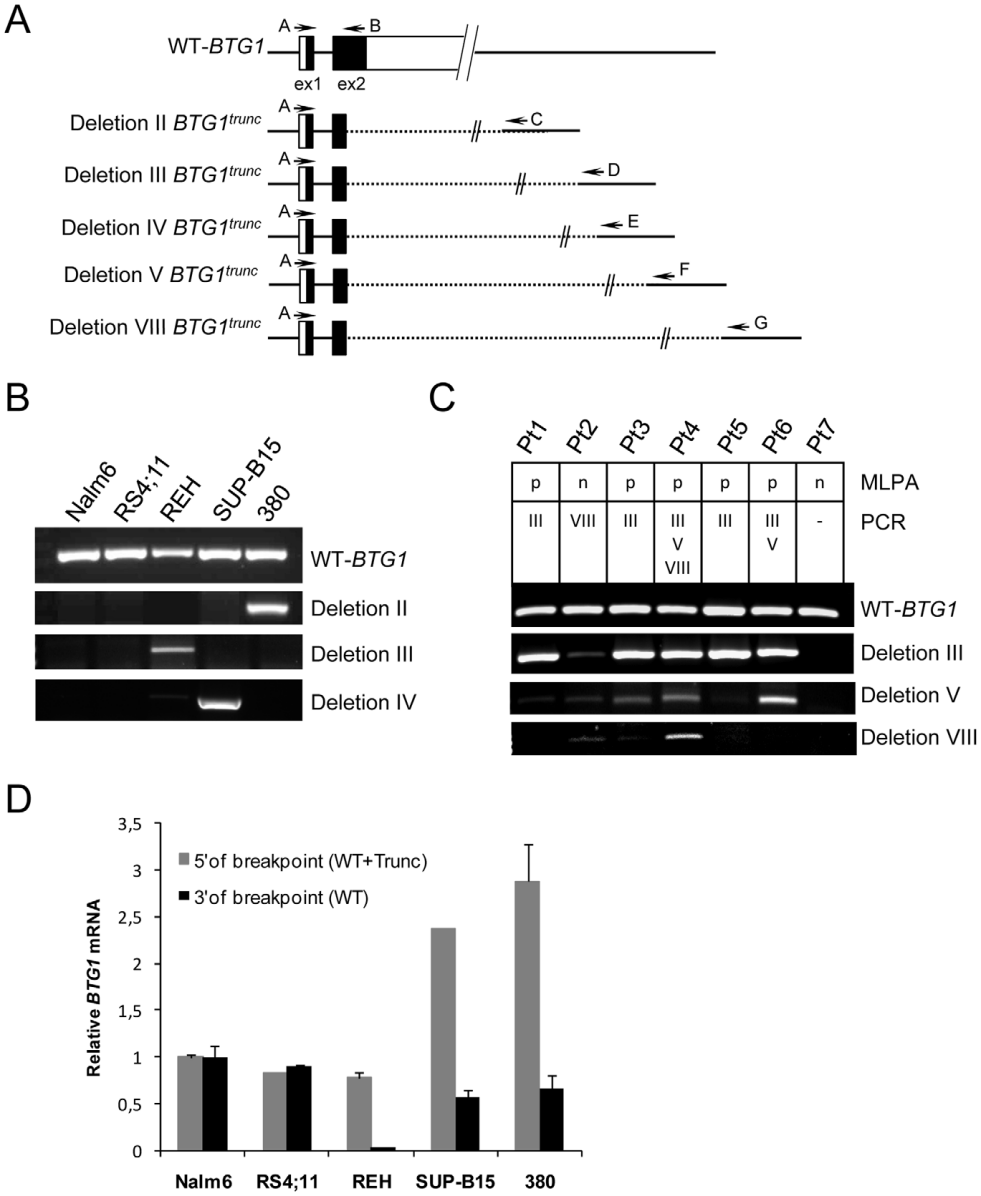
Expression of *BTG1* truncated read-through transcripts in BCP-ALL cells with *BTG1* deletions. (A) Schematic representation of the wild-type human *BTG1* gene, existing of two partly coding exons, and five different *BTG1* transcripts due to *BTG1* gene deletions. Exons are represented by black (coding) or white (non-coding) bars. Indicated are the RT-PCR primers that were used to detect expression of the wild-type *BTG1* transcript (primers A and B), or one of the *BTG1* truncated read-through transcripts for deletion II (primers A and C), deletion III (pimers A and D), deletion IV (primers A and E), deletion V (primers A and F), or deletion VIII (primers A and G). (B) RT-PCR analyses on total RNA isolated from the BCP-ALL cell lines Nalm6 and RS4;11 (*BTG1* wild-type) and REH, SUP-B15 and 380, each with distinct monoallelic *BTG1* deletions. (C) RT-PCR analyses on primary BCP-ALL samples in which a single *BTG1* deletion (Pt1, Pt2, Pt3 and Pt5), multiple *BTG1* deletions (Pt4 and Pt6) or no *BTG1* deletions were detected with genomic PCR (Pt7). Type of deletions (III, V, or VIII) and outcome of MLPA (p: deletion-positive; n: deletion-negative) are indicated. *BTG1* read-through transcripts were verified by sequencing (Table S5), except for Pt3-deletion V, which was an unrelated DNA sequence. (D) Quantitative real-time RT-PCR data representing relative expression levels of *BTG1* measured 5′ (primers exon 1/2) and 3′ of the *BTG1* breakpoint hotspot (primers exon 2). Expression levels were normalized to *HPRT* levels, and compared to the expression level in Nalm6, which was set to 1. The data shown represent the average of two independent cDNA reactions and triplicate qRT-PCR reactions.

Virtually all deletions affecting the *BTG1* locus target the second exon of *BTG1*, leaving the possibility of expression of a truncated protein product. To determine whether a truncated *BTG1* mRNA was expressed from the rearranged allele, RT-PCR using breakpoint-flanking primers was performed on three BCP-ALL cell lines with a *BTG1* deletion (380, REH and SUP-B15 with deletions II, III and IV, respectively) and two cell lines that are *BTG1* deletion negative (Nalm6 and RS4;11) (Figure 3). Wild-type *BTG1* mRNA was found to be present in all cell lines, whereas truncated read-through transcripts specific for each type of deletion could be detected only in the three deletion positive BCP-ALL cell lines (Figure 3B). Similarly, we detected *BTG1* read-through transcripts in primary BCP-ALL samples with *BTG1* deletions III, V and VIII, respectively, several of which could be confirmed on genomic DNA of these patients (Figure 3C). Notably, the presence of multiple *BTG1* deletion subclones as observed at the genomic level was also detectable at the transcript level and subsequent sequencing confirmed the unique identity of each clone (Table S5). Quantitative RT-PCR revealed that in REH, SUP-B15 and 380 cells the rearranged *BTG1* allele was expressed at significantly higher levels compared to the wild-type *BTG1* allele, which may reflect differences in mRNA stability (Figure 3D). These results indicate that monoallelic *BTG1* deletions result in the expression of truncated *BTG1* transcripts, encoding more than half of the BTG1 protein.

To assess the function of the truncated BTG1 protein, a C-terminal deletion variant of BTG1, which terminates at the common breakpoint after amino acid residue 100, was cloned in vector pcDNA3.1 and tested for protein expression. For comparison, wild-type *BTG1* was included in the analyses (Figure S1A). Full-length HA-tagged BTG1 protein is subject to proteosomal degradation [8], but in the presence of proteosome inhibitor MG132, expression could be readily detected (Figure S1B). In contrast, truncated BTG1 (HA-BTG1-Trunc) protein levels appeared to be highly unstable and hardly detectable even in the presence of MG132 (Figure S1B), while mRNA expression levels were equal to those of full-length HA-BTG1 (Figure S1C). Based on these findings, we conclude that the read-through transcripts that are expressed upon focal loss of *BTG1*, are unlikely to give rise to functionally active protein, which favors a BTG1 haploinsufficiency scenario.

### *BTG1* Deletions May Result from Illegitimate RAG-Mediated Recombination

Because illegitimate RAG-mediated recombination has been implicated in the origin of several translocations and deletions in leukemia [18]–[20], we examined the sequences flanking each of the *BTG1* breakpoints for the presence of recombination signal sequences (RSS). RSS motifs represent moderately conserved heptamer (CACAGTG) and nonamer (ACAAAAACC) sequences separated by either a spacer of 12±1 bp (12-RSS) or a spacer of 23±1 bp (23-RSS). The RAG complex only joins gene segments containing RSSs with different spacer lengths, complying to the 12/23 rule for efficient V(D)J recombination (Figure S2). Notably, we found that a 23-RSS was present at the recombination hotspot in the second exon of *BTG1*, while all but one of the centromeric breakpoints were found to harbor a 12-RSS (Table 3; Table S2). Furthermore, all deletion breakpoints contained a random addition of single nucleotides between the joining ends, which is most likely due to the action of terminal deoxynucleotidyl transferase (TdT). Together, these findings strongly support a role for illegitimate RAG-mediated recombination in the occurrence of *BTG1* deletions.

**Table 3:** Authentic RSSs and candidate RSSs flanking breakpoints of *BTG1* microdeletions.

RSS	Heptamer	Spacer	Nonamer
Optimal consensus	CACAGTG		ACAAAAACC
VκA27^a^	CACAGTG	12	ACAAAAACC
Jκ1^a^	CACAGTG b	23	ACAAAAACC
*TAL2* (9q32)^c^	CACTGTG b	13	ATAAAAATA
*LMO2* (11p13)^c^	CACAGTA b	12	GCAATAATT
*BTG1* exon 2	CACTCTG	23	ACAGAATTG
Deletion I	CACAGTA b	12	CCAGGACAT
Deletion III	CACAATG b	13	ACTGAAATG
Deletion IV	CACAGCT b	12	ACATTTTCA
Deletion V	CACTGCA b	12	GCAATAACC
Deletion VI	CACAGAG b	13	ACAATATAG
Deletion VII	CACTGTG b	12	ATATATTCT
Deletion VIII	CACAGTG b	12	ACAATTAAT

Mismatches from consensus are underlined;

a RSSs flanking V(D)J gene segments;

b Sequence shown is in reverse complement orientation;

c Functional cryptic RSSs at proto-oncogene breakpoints [18].

### The *BTG1* Gene Exhibits Elevated H3K4me3 Levels in B-Lineage Cells

**Figure 4:**
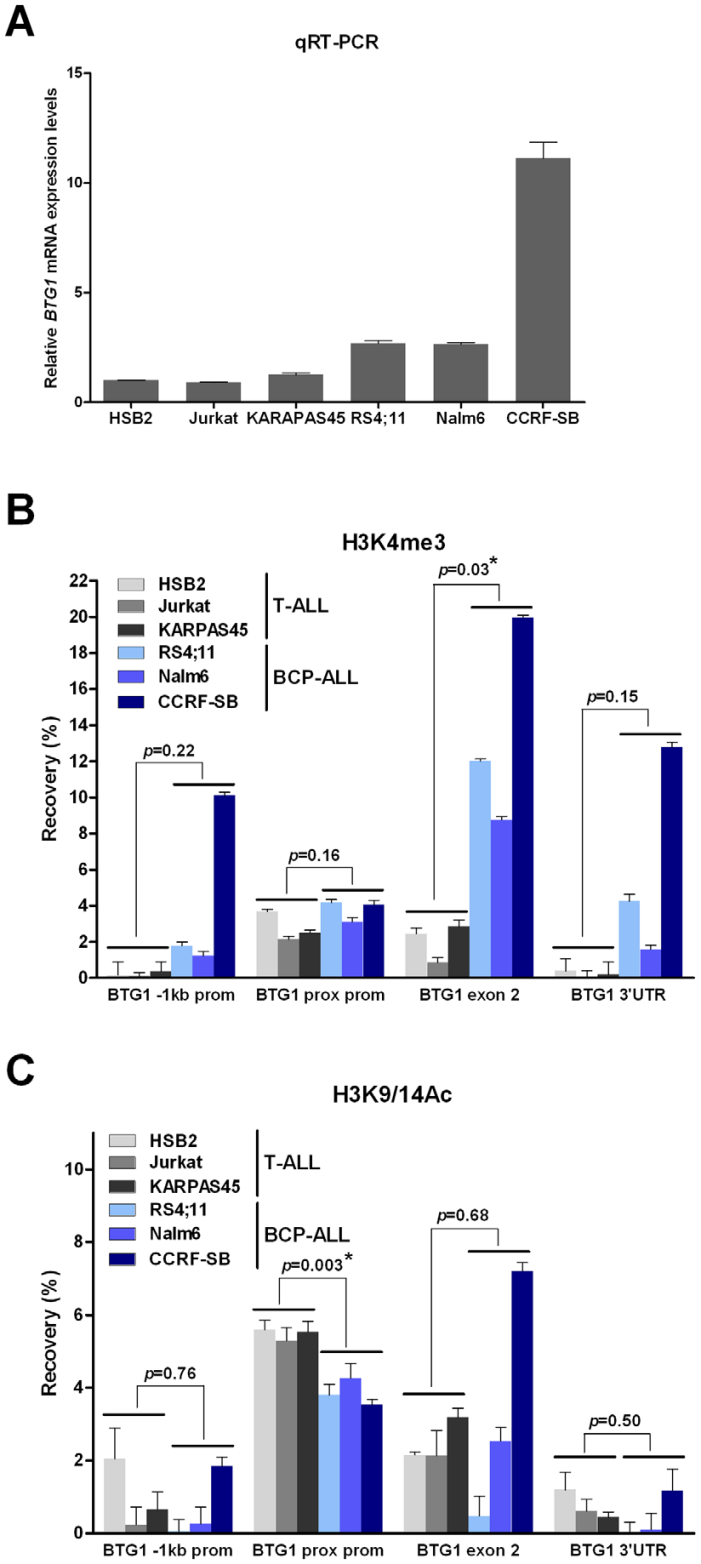
Increased levels of H3K4me3 at the *BTG1* locus in BCP-ALL versus T-ALL cell lines. (A) Quantitative real-time RT-PCR data representing relative expression levels of *BTG1* in T-ALL cell lines HSB2, Jurkat and KARPAS45, and BCP-ALL cell lines RS4;11, Nalm6 and CCRF-SB (*HPRT* normalized and related to HSB2 expression levels). Data shown are the average of two independent cDNA reactions and triplicate qRT-PCR reactions. (B and C) Percentage recovery after ChIP performed with H3K4me3 antibody (B) or H3K9/14Ac antibody (C) on T-ALL (HSB2, Jurkat, KARPAS45) and BCP-ALL (RS4;11, Nalm6 and CCRF-SB) cell lines. Real-time quantitative PCR was performed with primers specific for the region 1 kb upstream of the transcription start site (−1 kb prom), directly flanking the transcription start site (prox prom), the second exon near the breakpoint hotspot (exon 2) and towards the end of the 3′untranslated region (3′UTR) at the second (and last) exon of the human *BTG1* gene. Values represent two independent ChIP experiments. Student's *t*-test was performed to assess differences between the average recovery of T-ALL versus BCP-ALL samples. Asterisk (*) indicates a p-value<0.05.

Recent studies have shown that RAG-mediated V(D)J recombination is controlled by histone modifications, including histone H3 and H4 acetylation and H3 trimethylation, that are normally present at promoter regions of actively transcribed genes [26], [27]. More specifically, it has been demonstrated that the RAG2 plant homeodomain (PHD) finger binds directly to H3K4me3, thereby stimulating the catalytic activity of the RAG enzyme complex [28]. To reveal whether differences in levels of H3K4 trimethylation and/or H3K9/14 acetylation could explain the lineage-specific occurrence of *BTG1* deletions, chromatin immunoprecipitation (ChIP) experiments were performed targeting different positions within the *BTG1* gene in several B-lineage and T-lineage cell lines exhibiting abundant *BTG1* expression (Figure 4A). As expected, prominent levels of H3K4me3 were present at the proximal promoter region close to the transcription start site in both B-lineage and T-lineage cell lines (Figure 4B). In contrast, only BCP-ALL cell lines displayed significantly higher levels of H3K4me3 near the breakpoint hotspot within the second exon of *BTG1*. H3K9/14 acetylation levels were found to significantly differ at the proximal promoter region between B- and T-lineage cells, but not at the body of the *BTG1* gene (Figure 4C). In conclusion, our data suggest that illegitimate RAG-mediated recombination at the deletion breakpoint hotspot within the second exon of *BTG1* in B-lineage cells may be facilitated by increased levels of H3K4me3.

## Discussion

In this study, we demonstrate that *BTG1*, a recurrent target in pediatric ALL, is affected by gene truncating deletions, which occur predominantly in two cytogenetic subgroups of pediatric BCP-ALL, i.e., the *ETV6-RUNX1* positive (19%) and *BCR-ABL1* positive (26%) subgroups. Within these subgroups, *BTG1* deletions frequently arise independently in different subclones, which is in full conformity with the recently reported complex multi-clonal evolution model of ALL [24], [25]. In addition, our results suggest a role for *BTG1* deletions in the clonal selection and outgrowth in these BCP-ALL subgroups.

Through high-resolution genomic profiling, *BTG1* was only recently identified as a recurrent target in pediatric ALL [2]–[4]. Here we demonstrate, using a cohort of 831 pediatric ALL cases, that *BTG1* is targeted by a restricted number of well-demarcated genomic deletions. These microdeletions were found predominantly as monoallelic events at the clonal level in about 9% of the BCP-ALL cases, and were found to be completely absent in the T-ALL cases studied. Promoter hypermethylation events or sequence mutations were not detected. In contrast, *BTG1* mutations have frequently been found in diffuse large B-cell lymphoma, arguing that other mutation mechanisms may target *BTG1* in more maturated B-lineage malignancies [10]. Eight different deletions in 52 cases were molecularly cloned and sequenced. Together, the three most prevalent deletions (III, V and VIII) were found in 81% of the total. Of note, the vast majority of telomeric *BTG1* deletion breakpoints were found to be tightly clustered within a stretch of 10 bp in the last exon of *BTG1.*

This remarkable finding has both structural and functional implications. Structurally, this breakpoint clustering implies the presence of sequence features underlying the origin of *BTG1* deletions. Virtually all breakpoints that were mapped, including those within exon 2, were flanked by non-canonical RSS sequences, which strongly suggests that they have arisen from illegitimate RAG-mediated recombination. In line with these observations, BCP-ALL derived cell lines showed local accumulation of H3K4me3 within the coding region of the *BTG1* gene including the second exon, which harbors the breakpoint hotspot. This epigenetic mark is normally associated with the proximal promoter of actively transcribed genes and, in addition, has been shown to act as a docking site for RAG2 binding, thereby facilitating V(D)J recombination [28]. In contrast, in T-ALL-derived cell lines these H3K4me3 marks were not enriched at the site of the *BTG1* gene body, which indicates that in these cells the locus is less accessible for the RAG enzyme complex. Illegitimate recombination events at cryptic RSS motifs have been implicated in several recurrent chromosomal abnormalities in lymphoid malignancies, such as translocations and deletions. For example, both *ETV6-RUNX1* and *BCR-ABL1* translocations have been suggested to arise from mistargeting of RAG proteins that facilitate RAG-mediated transposition or at least create one of the initiating lesions in the form of nicks [29]–[31]. This mistargeting of RAG proteins may occur as a rare spontaneous event during lymphocyte differentiation, but may also result from increased or prolonged RAG activity in leukemic lymphoid blasts. This latter phenomenon may explain the subtype specificity of *BTG1* deletions and/or the co-occurrence of these deletions with *ETV6-RUNX1* and *BCR-ABL1* translocations.

The recurrence of truncating *BTG1* deletions in BCP-ALL may also point towards a functional role in leukemogenesis. *BTG1* belongs to the *BTG/TOB* family of anti-proliferative genes (*BTG1*-*BTG4*, *TOB1* and *TOB2)* implicated in several types of cancer [9]. We recently reported that in BCP-ALL, BTG1 regulates the glucocorticoid receptor (GR)-dependent transcriptional response in leukemia cells, while loss of BTG1 expression leads to glucocorticoid resistance in cell line models [8]. In addition, we have shown that leukemia clones carrying *BTG1* deletions can survive therapy and rearise as a predominant clone in relapse or, as shown also by others, can occur as new lesions in relapse [21], [32]. Many *BTG1* deletions result in almost identical truncations of the open reading frame and the consequent loss of two conserved C-terminal protein interaction domains. Nevertheless, dominant-negative or gain-of-function effects of the truncated BTG1 protein are less likely since the truncated BTG1 protein appears to be highly unstable. Furthermore, we also encountered deletions encompassing the entire open reading frame or only part of the 3′-UTR, indicating that truncation of the *BTG1* open reading frame is highly frequent but not essential.

The fact that *BTG1* deletion breakpoints are tightly clustered provides an opportunity to perform rapid and sensitive deletion screening through breakpoint-spanning PCRs. By applying this screening approach to our pediatric ALL cohort we identified, besides the most prominent *BTG1* deletions, additional deletions in a substantial fraction (18%) of BCP-ALL cases at the subclonal level. These deletions remained undetected using standard procedures like MLPA or SNP arrays. In several cases, multiple deletions co-occurred in a single patient, each carrying unique breakpoint-spanning sequences that were detectable both at the genomic and at the transcript level. Analogous to the most prominent deletions, these subclonal lesions displayed a very similar distribution over different cytogenetic subgroups in BCP-ALL and were not present in T-ALL or normal bone marrow samples. Moreover, in one patient, this subclonal *BTG1* deletion-positive clone at diagnosis reappeared as the major clone at relapse, whereas in relapse samples of five other patients new (sub)clonal *BTG1* deletions appeared. These findings are completely in line with recent studies describing the clonal evolution of *ETV6-RUNX1* and *BCR-ABL1* positive ALLs with a clonal evolution during leukemia development showing that, upon treatment, recurrent lesions occur repeatedly and independently within a single patient, giving rise to a complex variety of slightly different subclones [24], [25]. Our current data provide additional evidence for this model at the molecular level by showing that *BTG1* deletions arise independently in multiple ALL subclones in a context-dependent manner.

In addition, our results provide insight into the genetic lesions that act in concert with *BTG1* deletions in BCP-ALL development. We found, for example, that deletions in *ETV6*, *EBF1* and *RB1* co-occur with *BTG1* deletions, indicating that loss of normal BTG1 function may add to defects in pRb/E2F-mediated cell cycle and EBF1-mediated B-cell differentiation pathways. Illegitimate RAG-mediated recombination has been suggested as the responsible mechanism for *IKZF1* [19], [20], *CDKN2A/B* [33], [34] and *LMO2* [35] deletions, as well as *ETV6-RUNX1* translocations [30]. It remains to be established whether RAG-mediated recombination, or other mechanisms like CSR, are implicated in the occurrence of *RB1* and *EBF1* deletions. However, similar to *BTG1*, these recurrent gene deletions may repeatedly arise in different subclones due to local subtype-specific accessibility of these loci to specific recombination machineries, followed by clonal selection and outgrowth.

In conclusion, our comprehensive analysis of *BTG1* aberrations revealed that this gene is recurrently and exclusively affected by deletions in specific BCP-ALL subtypes. *BTG1* deletions can arise independently in different ALL subclones, which either develop into a predominant clone at diagnosis, remain present as minor subclones during the course of the disease, or develop into the major clone at relapse. As such, this phenomenon may provide a molecular explanation for the model of multiclonal evolution during leukemogenesis.

## Materials and Methods

### Clinical Samples

A total of 831 patients diagnosed with BCP-ALL (n = 722) and T-ALL (n = 109) were included in this study. Diagnosis samples (n = 831) and matched relapse samples (n = 62) were collected by the Dutch Childhood Oncology Group (DCOG) and the Radboud University Nijmegen Medical Centre, the Netherlands. Mononuclear cells were harvested through Ficoll gradient separation and DNA was isolated using a QiaAmp purification kit (Qiagen, Venlo, The Netherlands). DNA from non-leukemic bone marrow samples was provided by Dr. Joop Jansen (Department of Hematology, Radboud University Nijmegen Medical Centre, the Netherlands). Written informed consent was obtained for all patient and control samples.

### Cell Lines

The human BCP-ALL (CCRF-SB, RS4;11, Nalm6, REH, SUP-B15, 380, 697, SEM, TOM1, MUTZ5) and T-ALL cell lines (MOLT4, Jurkat) were purchased either from ATCC or DSMZ. T-ALL cell lines CML-T1, HSB2, and KARPAS45 were obtained from Dr. Adolfo Ferrando (Columbia University, NY, USA) and T-ALL cell line TK6 was obtained from Dr. Albert Fornace (NCI, Bethesda, USA). Leukemia cell lines were maintained in RPMI-1640 medium (Invitrogen) supplemented with 10% fetal calf serum, 100 U/mL penicillin sodium, and 100 µg/mL of streptomycin sulfate at 37°C in a humidified air atmosphere containing 5% carbon dioxide.

### Multiplex Ligation-Dependent Probe Amplification

Leukemic patient samples and cell lines (380, 697, CCRF-SB, RS4;11, SEM, TOM1, REH, MUTZ5, SUP-B15, Nalm6, Jurkat, KARPAS45, MOLT4 and TK6) were analyzed for copy number changes in *BTG1* using multiplex ligation-dependent probe amplification (MLPA). DNA was isolated using a QiaAmp purification kit (Qiagen). A total of 9 probes was developed using MeltIngeny software and guidelines provided by MRC-Holland (Amsterdam, The Netherlands; Table S6). MLPA analyses were performed using SALSA MLPA reaction mixture and P200-A1 reference probe-mix (MRC Holland) as previously described [5]. In addition, we determined the copy number status of several leukemia-associated genes (*PAX5*, *IKZF1*, *EBF1*, *CDKN2A*, *CDKN2B*, *RB1*, *ETV6*, *BTG1*) with the SALSA MLPA kit P335-A2 ALL-IKZF1 (MRC Holland) according to manufacturer's instructions.

### Bisulfite Sequencing

In 25 BCP-ALL patient diagnosis samples, we determined the methylation status of the *BTG1* promoter using bisulfite sequencing. A total of 500 ng DNA per sample was bisulphite converted using the EZ DNA Methylation™ Kit (Zymo research corporations, Leiden, The Netherlands) according to manufacturer's protocol. Subsequently, the CpG island was PCR amplified (for primers see Table S7), cloned in a pGEM-T vector (Promega Benelux BV, Leiden, The Netherlands), transformed in competent *E. Coli* DH5α cells and plated on an LB agar plate containing 7 µg/ml ampicillin, 200 nM Isopropyl β-D-1-thiogalactopyranoside (IPTG) and 200 µg/ml X-Gal. After overnight incubation, individual white clones were selected for colony PCR and sequenced using a 3730 Sequence Analyzer (Applied Biosystems, Foster City, CA, USA). Sequences were analyzed using Vector NTI software (Advance TM 11.0, release December 15 2008, Invitrogen, Breda, the Netherlands). In vitro methylated DNA was used as a positive control. Briefly, 1 µg DNA was incubated with 4 U M.SssI CpG Methyltransferase (New England Biolabs, Ipswich, United Kingdom), 160 µM S-adenosylmethionine, 50 mM NaCl, 10 mM Tris-HCl, 10 mM MgCl_2_ and 1 mM Dithiothreitol (pH7.9) for 4 hours at 37°C. As a negative control, HEK293 DNA was amplified by using the GenomePlex Complete Whole Genome Amplification Kit (Sigma-Aldrich, Zwijndrecht, The Netherlands), according to manufacturer's protocol.

### *BTG1* Mutation Detection

Sequencing of the first (n = 135) and second (n = 337) exon of the *BTG1* gene was performed on PCR-amplified exons using flanking intron-based primers (listed in Table S7). PCR amplifications were performed with 10 ng DNA, 200 nM of each primer, 2 (ex1) or 2.5 (ex2) mM MgCl_2_, 200 µM dNTPs, 1 M GC melt (Clontech, Saint-Germain-en-Laye, France), 10× PCR buffer II, 2.5 U AmpliTaq Gold (Applied Biosystems) using 5 minutes preheating at 96°C, followed by 35 cycles of 30 seconds at 96°C, 30 seconds at 55.3°C and 1 min at 72°C, and termination for 3 min at 72°C. The samples were sequenced using a 3730 Sequence Analyzer (Applied Biosystems) and the results were analyzed using Vector NTI software (Invitrogen).

### Genomic SNP Arrays

To characterize the *BTG1* deletions in further detail, we performed high-resolution copy number and genotyping analyses using Affymetrix SNP6.0 arrays according to standard protocols provided by the manufacturer. Genotypes were generated using Affymetrix Genotyping Console v2.1 and Nexus 5.0 software (BioDiscovery, Inc 2010, build version 4621). Normal copy number variation was filtered out using data from our in-house database (containing over 600 healthy control samples), Hapmap, and the Database of Genomic Variants (http://projects.tcag.ca/variation/).

### Breakpoint Mapping and PCR-Based Validation

Starting from the SNP array data, we used a stepwise approach to zoom in on eight individual breakpoints of different *BTG1* deletions. We determined the exact genomic breakpoints and interstitial sequences using Q-PCR, long-range PCR and finally, standard PCR and sequencing. Q-PCR was performed on a 7900HT FAST Real-Time PCR system (Applied Biosystems) with SYBR Green to detect PCR product. Long range PCR was performed using TaKaRa La Taq (Takara Bio Inc, Otsu, Shiga, Japan). PCR amplicons were sequenced using a 3730 Sequence Analyzer (Applied Biosystems) and analyzed with VectorNTI software (Invitrogen). The primer pairs used to detect and sequence the eight different breakpoints are listed in Table S7. For PCR-based validation we used 20 ng DNA, 200 nM of each primer, 2 or 2.5 mM MgCl_2_, 200 µM dNTPs, 10× PCR buffer II, 2.5 U AmpliTaq Gold (Applied Biosystems) using 5 minutes preheating at 95°C, followed by 10 cycles of 30 seconds at 95°C, 2 min at 60°C or 57.8°C and 2 min at 72°C, and a further 30 cycles of 30 seconds at 95°C, 30 seconds at 60°C or 57.8°C and 2 min at 72°C, and termination for 10 min at 72°C.

### RT–PCR

Total RNA from the BCP-ALL and T-ALL cell lines was extracted with Trizol (Invitrogen), while total RNA from diagnosis patient samples was isolated using the RNeasy kit (Qiagen), both according to the manufacturer's instructions. Total RNA was converted to cDNA via RT-PCR using random 6-mers and Superscript III Reverse Transcriptase (Invitrogen). Thereafter, cDNA was resolved in 50 µl ddH_2_O, and PCR amplifications were performed in 30 µl reactions at standard concentrations (1.5 mM MgCl_2_, 0.2 mM dNTP, 1× PCR buffer (Invitrogen), 2 U Platinum Taq (Invitrogen), 0.3 µM of each primer, and 2 µl cDNA template) (see Table S7 for primer sequences). PCR reactions were performed for 35 cycles at an annealing temperature of 58°C using an Eppendorf Mastercycler. The PCR fragments obtained were resolved by 1% agarose gel electrophoresis, isolated using Qiagen gel extraction kit and cloned into pGEM-T easy for sequencing with M13 primers. Quantitative real-time PCR was performed to determine the expression of *BTG1* mRNA sequences 5′ to the breakpoint hotspot (exon primers flanking intron 1), *BTG1* exon 2 (primers flanking breakpoint hotspot) and HPRT with Power SYBR Green (Applied Biosystems) (Table S7). Reactions were performed in triplicate on two independent cDNA templates using an Applied Biosystems 7500 Real-Time PCR thermocycler (ABI). Data were represented as fold differences relative to *BTG1* mRNA expression in Nalm6 cells normalized to HPRT expression based on calculation of 2^−ΔΔCt^.

### Cloning and Protein Expression Analyses *BTG1* Constructs

HA-BTG1-Trunc was generated by PCR based on full-length HA-BTG1 [8], using primers indicated in Table S7. PCR products were purified, cloned into the *EcoRI* and *XhoI* sites of pcDNA3.1 (Invitrogen) and verified by Sanger sequencing. To analyze protein expression of the different BTG1 constructs, HEK293 cells were transfected with 10 µg of plasmid DNA using linear Polyethyleneimine (PEI, Polysciences, Warrington, PA). The next day cells were splitted over two dishes and, 24 hours after transfection, treated with 5 µM MG132 (Peptides International, Louisville, KY) in DMSO or vehicle for 16 hours. For detection of HA-BTG1 protein expression, 5×10^6^ cells were lysed in Laemmli sample buffer containing 2% SDS and 100 mM DTT, boiled for 5 minutes, loaded on 15% SDS-PAGE, blotted on PVDF membrane, and stained with HA antibody, clone 3F10 (Roche Diagnostics). Protein expression was visualized using ECL Plus Western Blot Detection System (GE Healthcare) and FluorchemE digital imaging device (Cell Biosciences).

### Chromatin Immunoprecipitations

After crosslinking for 15 min with 1% formaldehyde, the leukemia cells were re-suspended in ice-cold lysis buffer (50 mM HEPES [pH7.6], 140 mM NaCl, 1 mM EDTA, 1% Triton X-100, 0.1% Sodium Deoxycholate) supplemented with Complete protease inhibitors (Roche) at a density of 35×10^6^ cells/mL and sonicated for 15 min, 30 seconds on and 30 seconds off (Bioruptor Diagenode). Chromatin was incubated overnight in the presence 0.1% BSA, 20 mM HEPES (pH 7.6), 150 mM NaCl, 1 mM EDTA, 0.5 mM EGTA, 0.15% SDS, 1% Triton X-100, protein A/G beads (Santa Cruz), Complete protease inhibitors and 2 µg H3K4me3 or H3K9/14Ac antibody (both from Diagenode). Beads were washed two times in buffer 1 (0.1% SDS, 0.1% Sodium Deoxycholate, 1% Triton X-100, 150 mM NaCl, 1 mM EDTA, 0.5 mM EGTA, 20 mM HEPES [pH7.6]), one time in buffer 2 (0.1% SDS, 0.1% Sodium Deoxycholate, 1% Triton X-100, 500 mM NaCl, 1 mM EDTA, 0.5 mM EGTA, 20 mM HEPES [pH7.6]), one time in buffer 3 (0.5% Sodium Deoxycholate, 0.5% NP40, 250 mM LiCl , 1 mM EDTA, 0.5 mM EGTA, 20 mM HEPES [pH7.6]), and two times in buffer 4 (1 mM EDTA, 0.5 mM EGTA, 20 mM HEPES [pH 7.6]). Chromatin was eluted from the beads with 1% SDS/100 mM NaHCO_3_ and de-crosslinked together with input material for 4 hrs at 65°C with 200 mM NaCl. After phenol∶chloroform∶isoamylalcohol extraction, DNA was precipitated in the presence of 10 µg glycogen. Subsequently, diluted DNA was subjected to quantitative PCR using Power SYBR Green (Applied Biosystems) and an Applied Biosystems 7500 Real-Time PCR thermocycler (primers are listed in Table S7). The percentage recovery was equal to dilution factor of input DNA×2^(Ct Input-Ct ChIP)^×100.

### Statistical Analyses

Co-occurrence of copy number alterations and cytogenetic subgroups was compared using crosstabs and a standard chi-square test or Fisher's exact test when sample groups were small. The differences between H3K4me3 and H3K9/14Ac levels in T-ALL versus BCP-ALL samples was assessed using a Student's *t*-test. Statistical analyses were carried out using the SPSS statistical package (IBM, Chicago, IL, USA; release 16.0.2, April 2008) and two-sided P-values below 0.05 were considered to be statistically significant.

## Supporting Information

Figure S1 BTG1-Trunc protein levels are highly unstable and expressed at extremely low levels. (A) Schematic representation of the full length BTG1 protein and deletion mutant BTG1-Trunc, which mimics the common deletion variant observed in BCP-ALL harboring monoallelic *BTG1* deletions. The conserved BoxA, BoxB and BoxC domains as well as the nuclear hormone receptor interaction residues LxxLL are indicated. (B) Immunoblot shows BTG1 protein levels from pcDNA3.1 vectors expressing HA-BTG1 (WT) and HA-BTG1-Trunc (Tr) upon transfection in HEK293 cells in the absence or presence of 5 µM MG132 for 16 hrs. Protein expression is detected with HA antibody 3F10. (C) *BTG1* RNA expression levels in the same pool of transfected HEK293 cells: Real-time PCR was performed on cDNA generated in the absence (black bars) or presence (grey bars) of Reverse Transcriptase (RT). *BTG1* expression was normalized to expression levels of the basal transcription factor TBP.
(PDF)

Figure S2 Monoallelic *BTG1* deletions result from aberrant RAG-mediated recombination. The recombination signal sequence (RSS) consists of a conserved 7-bp sequence (heptamer; consensus CACAGTG), a 9-bp sequence (nonamer consensus ACAAAAACC), and an intervening, non-conserved 12±1 or 23±1 bp spacer sequence. Recombination that takes place between RSS found in the opposite chromosomal orientation will result in deletion of the intervening DNA sequences. Joining of the RAG-mediated double-strand breaks is carried out by the non-homologous DNA end-joining (NHEJ) proteins via the concerted action of the Ku70/80 proteins, DNA-dependent protein kinase (DNA-PK), Artemis, XRCC4, DNA Ligase 4 (Lig4) and terminal deoxynucleotidyl transferase (TdT). RAG-mediated recombination results in the formation of a precise signal joint and modified coding joint. (A) Schematic representation of the human B cell receptor IgH locus, with the number of gene segments indicated above the V, D and J gene loci. V(D)J recombination occurs only between two gene segments flanked, respectively, by 12-bp RSS and a 23-bp RSS, referred to the 12/23 rule. Thus, the 12/23 rule prohibits direct V_H_-to-J_H_ joining. (B) Schematic representation of the human *BTG1* gene on chromosome 12, which contains a 23-bp RSS at the deletion hotspot in the second exon, and one of the seven identified distal deletion breakpoints harboring a 12-bp RSS. RAG-induced recombination results in deletion of the intervening DNA and a modified coding joint at the *BTG1* gene, where TdT adds random, non-templated nucleotides.
(PDF)

Table S1 *BTG1* microdeletions co-occur with deletions in recurrently affected genes in BCP-ALL. ^a^Because of missing values, numbers do not always add up to 722 BCP-ALL cases.
(PDF)

Table S2 *BTG1* deletion breakpoint sequences in BTG1 MLPA deletion-positive BCP-ALL cases and cell lines. Sequencing of intragenic *BTG1* deletions demonstrates the presence of (near) consensus DNA sequence motifs for V(D)J recombination flanking the breakpoint hotspot in exon 2 of *BTG1* and the distal breakpoint clusters. The consensus heptamer RSS [CAC(A/T)(A/G)(C/T)(A/G) on (+) strand and (C/T)(A/G)(C/T)(A/T)GTG on (-) strand] is shown in red and bold. Mismatches from consensus are underlined. Single nucleotide mutations are indicated in grey. The nucleotides inserted between the proximal *BTG1* and the distal breakpoints are indicated.
(PDF)

Table S3 Number of unique *BTG1* deletion-spanning sequences in the BTG1 MLPA deletion-positive BCP-ALL cases and cell lines.
(PDF)

Table S4 Subclonal *BTG1* microdeletion occurrence within the cytogenetic subgroups. All patients screened were *BTG1* deletion-negative as determined by MLPA. Subclonal *BTG1* deletion status was determined using the PCR-based detection of deletions III, V and VIII. ^a^Because of missing values, numbers do not always add up to 89 BCP-ALL cases. Data was available for 77 cases on hyperdiploidy; 41 cases for *ETV6-RUNX1*; 60 cases for *BCR-ABL1;* 74 cases for *MLL*-rearrangement. ^b^The other subgroup encompasses cases negative for *ETV6-RUNX1*, *MLL*, *BCR-ABL1* translocations and/or hyperdiploidy. This group does not contain any *E2A-PBX1* translocation cases. ^c^Subgroup unknown includes all cases in which no data is available in one or more cytogenetic subgroups. ^d^Fisher's exact test was used when sample groups were small.
(PDF)

Table S5 *BTG1* fusion transcript sequences detected in independent subclones. Deletion spanning sequence was confirmed by sequencing of genomic DNA of the same case.
(PDF)

Table S6 MLPA probes for copy-number analysis of the *BTG1* gene region. Universal M13 PCR primers are indicated in bold.
(PDF)

Table S7 Primers used for *BTG1* bisulfite sequencing, mutation screening, breakpoint mapping, expression analysis and CHIP assay.
(PDF)
