# Supplementary material for: The Origin and Nature of Tightly Clustered BTG1 Deletions in Precursor B-Cell Acute Lymphoblastic Leukemia Support a Model of Multiclonal Evolution
Source: PLoS Genet. 2012 Feb 16;8(2):e1002533. doi: 10.1371/journal.pgen.1002533 (PMC3280973; doi:10.1371/journal.pgen.1002533)
Supplement: Figure S1 — BTG1-Trunc protein levels are highly unstable and expressed at extremely low levels. (A) Schematic representation of the full length BTG1 protein and deletion mutant BTG1-Trunc, which mimics the common deletion variant observed in BCP-ALL harboring monoallelic BTG1 deletions. The conserved BoxA, BoxB and BoxC domains as well as the nuclear hormone receptor interaction residues LxxLL are indicated. (B) Immunoblot shows BTG1 protein levels from pcDNA3.1 vectors expressing HA-BTG1 (WT) and HA-BTG1-Trunc (Tr) upon transfection in HEK293 cells in the absence or presence of 5 µM MG132 for 16 hrs. Protein expression is detected with HA antibody 3F10. (C) BTG1 RNA expression levels in the same pool of transfected HEK293 cells: Real-time PCR was performed on cDNA generated in the absence (black bars) or presence (grey bars) of Reverse Transcriptase (RT). BTG1 expression was normalized to expression levels of the basal transcription factor TBP. (PDF) [file pgen.1002533.s001.pdf]

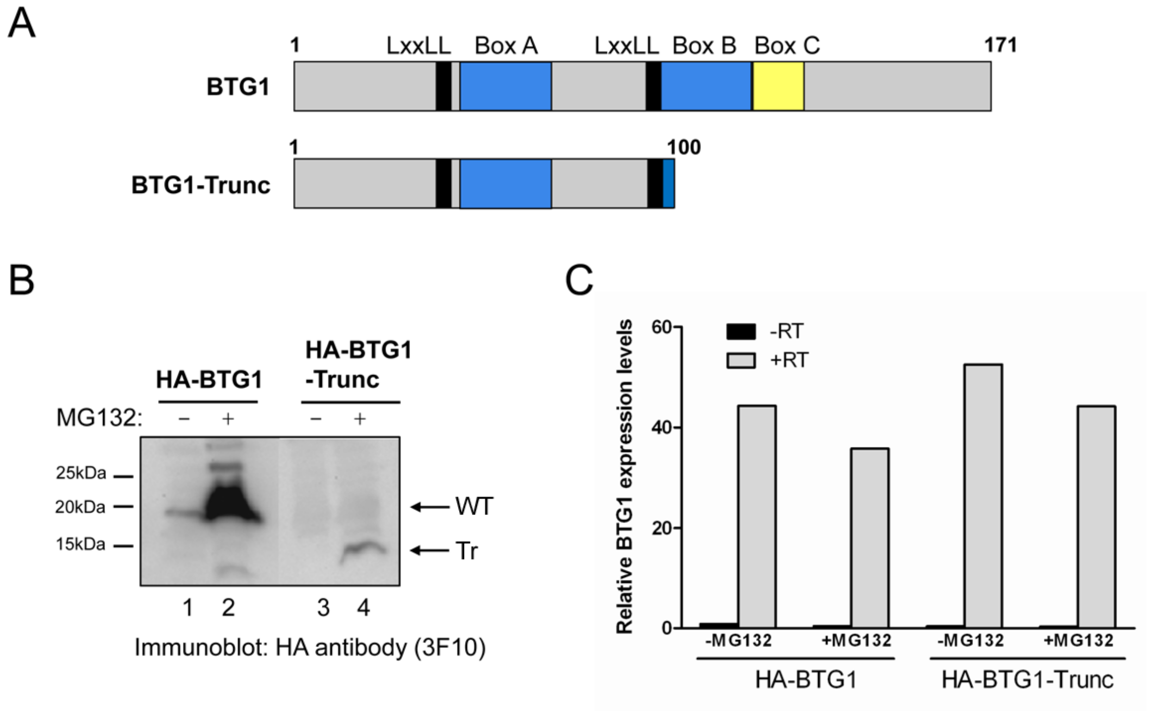

Figure S1. BTG1-Trunc protein levels are highly unstable and expressed at extremely low levels. (A) Schematic representation of the full length BTG1 protein and deletion mutant BTG1-Trunc, which mimics the common deletion variant observed in BCP-ALL harboring monoallelic *BTG1* deletions. The conserved BoxA, BoxB and BoxC domains as well as the nuclear hormone receptor interaction residues LxxLL are indicated. (B) Immunoblot shows BTG1 protein levels from pcDNA3.1 vectors expressing HA-BTG1 (WT) and HA-BTG1-Trunc (Tr) upon transfection in HEK293 cells in the absence or presence of 5 μM MG132 for 16 hrs. Protein expression is detected with HA antibody 3F10. (C) *BTG1* RNA expression levels in the same pool of transfected HEK293 cells: Real-time PCR was performed on cDNA generated in the absence (black bars) or presence (grey bars) of Reverse Transcriptase (RT). *BTG1* expression was normalized to expression levels of the basal transcription factor TBP.
