# Supplementary material for: The Origin and Nature of Tightly Clustered BTG1 Deletions in Precursor B-Cell Acute Lymphoblastic Leukemia Support a Model of Multiclonal Evolution
Source: PLoS Genet. 2012 Feb 16;8(2):e1002533. doi: 10.1371/journal.pgen.1002533 (PMC3280973; doi:10.1371/journal.pgen.1002533)
Supplement: Figure S2 — Monoallelic BTG1 deletions result from aberrant RAG-mediated recombination. The recombination signal sequence (RSS) consists of a conserved 7-bp sequence (heptamer; consensus CACAGTG), a 9-bp sequence (nonamer consensus ACAAAAACC), and an intervening, non-conserved 12±1 or 23±1 bp spacer sequence. Recombination that takes place between RSS found in the opposite chromosomal orientation will result in deletion of the intervening DNA sequences. Joining of the RAG-mediated double-strand breaks is carried out by the non-homologous DNA end-joining (NHEJ) proteins via the concerted action of the Ku70/80 proteins, DNA-dependent protein kinase (DNA-PK), Artemis, XRCC4, DNA Ligase 4 (Lig4) and terminal deoxynucleotidyl transferase (TdT). RAG-mediated recombination results in the formation of a precise signal joint and modified coding joint. (A) Schematic representation of the human B cell receptor IgH locus, with the number of gene segments indicated above the V, D and J gene loci. V(D)J recombination occurs only between two gene segments flanked, respectively, by 12-bp RSS and a 23-bp RSS, referred to the 12/23 rule. Thus, the 12/23 rule prohibits direct VH-to-JH joining. (B) Schematic representation of the human BTG1 gene on chromosome 12, which contains a 23-bp RSS at the deletion hotspot in the second exon, and one of the seven identified distal deletion breakpoints harboring a 12-bp RSS. RAG-induced recombination results in deletion of the intervening DNA and a modified coding joint at the BTG1 gene, where TdT adds random, non-templated nucleotides. (PDF) [file pgen.1002533.s002.pdf]

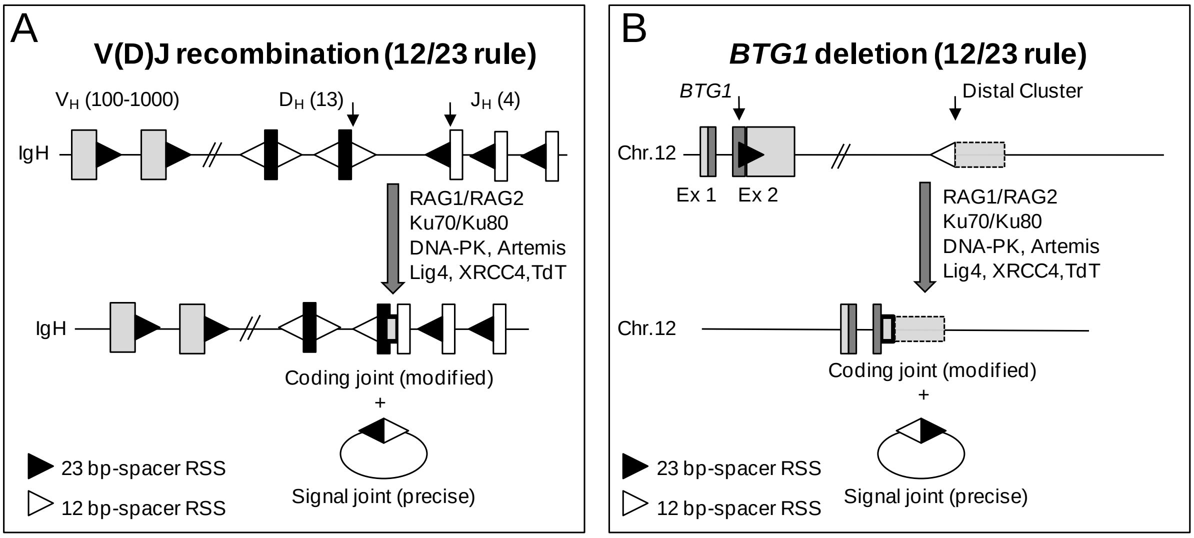

Figure S2. Monoallelic *BTG1* deletions result from aberrant RAG-mediated recombination. The recombination signal sequence (RSS) consists of a conserved 7-bp sequence (heptamer; consensus CACAGTG), a 9-bp sequence (nonamer consensus ACAAAAACC), and an intervening, non-conserved 12±1 or 23±1 bp spacer sequence. Recombination that takes place between RSS found in the opposite chromosomal orientation will result in deletion of the intervening DNA sequences. Joining of the RAG-mediated double-strand breaks is carried out by the non-homologous DNA end-joining (NHEJ) proteins via the concerted action of the Ku70/80 proteins, DNA-dependent protein kinase (DNA-PK), Artemis, XRCC4, DNA Ligase 4 (Lig4) and terminal deoxynucleotidyl transferase (TdT). RAG-mediated recombination results in the formation of a precise signal joint and modified coding joint. (A) Schematic representation of the human B cell receptor IgH locus, with the number of gene segments indicated above the V, D and J gene loci. V(D)J recombination occurs only between two gene segments flanked, respectively, by 12-bp RSS and a 23-bp RSS, referred to the 12/23 rule. Thus, the 12/23 rule prohibits direct V_H-to-J_H joining. (B) Schematic representation of the human *BTG1* gene on chromosome 12, which contains a 23-bp RSS at the deletion hotspot in the second exon, and one of the seven identified distal deletion breakpoints harboring a 12-bp RSS. RAG-induced recombination results in deletion of the intervening DNA and a modified coding joint at the *BTG1* gene, where TdT adds random, non-templated nucleotides.
