# Supplementary material for: The Origin and Nature of Tightly Clustered BTG1 Deletions in Precursor B-Cell Acute Lymphoblastic Leukemia Support a Model of Multiclonal Evolution
Source: PLoS Genet. 2012 Feb 16;8(2):e1002533. doi: 10.1371/journal.pgen.1002533 (PMC3280973; doi:10.1371/journal.pgen.1002533)
Supplement: Table S1 — BTG1 microdeletions co-occur with deletions in recurrently affected genes in BCP-ALL. aBecause of missing values, numbers do not always add up to 722 BCP-ALL cases. (PDF) [file pgen.1002533.s003.pdf]

Table S1. *BTG1* microdeletions co-occur with deletions in recurrently affected genes in BCP-ALL.

	Number of Samples[a]	*BTG1* deletion positive cases (%)	*BTG1* deletion negative cases (%)	P-value Chi-square
EBF1				<0.001
Deletion	41	11 (26.8)	30 (73.2)	
No deletion	671	53 (7.9)	618 (92.1)	
ETV6				0.007
Deletion	162	25 (15.4)	137 (84.6)	
No deletion	470	38 (8.1)	432 (91.9)	
RB1				<0.001
Deletion	60	14 (23.3)	46 (76.7)	
No deletion	567	48 (8.5)	519 (91.5)	
IKZF1				0.154
Deletion	104	13 (12.5)	91 (87.5)	
No deletion	609	50 (8.2)	559 (91.8)	
PAX5				0.715
Deletion	158	15 (9.5)	143 (90.5)	
No deletion	549	47 (8.6)	502 (91.4)	
CDKN2A				0.307
Deletion	166	20 (12.0)	146 (88.0)	
No deletion	474	44 (9.3)	430 (90.7)	
CDKN2B				0.173
Deletion	165	21 (12.7)	144 (87.3)	
No deletion	476	43 (9.0)	433 (91.0)	

[a]Because of missing values, numbers do not always add up to 722 BCP-ALL cases
