# Supplementary material for: The Origin and Nature of Tightly Clustered BTG1 Deletions in Precursor B-Cell Acute Lymphoblastic Leukemia Support a Model of Multiclonal Evolution
Source: PLoS Genet. 2012 Feb 16;8(2):e1002533. doi: 10.1371/journal.pgen.1002533 (PMC3280973; doi:10.1371/journal.pgen.1002533)
Supplement: Table S2 — BTG1 deletion breakpoint sequences in BTG1 MLPA deletion-positive BCP-ALL cases and cell lines. Sequencing of intragenic BTG1 deletions demonstrates the presence of (near) consensus DNA sequence motifs for V(D)J recombination flanking the breakpoint hotspot in exon 2 of BTG1 and the distal breakpoint clusters. The consensus heptamer RSS [CAC(A/T)(A/G)(C/T)(A/G) on (+) strand and (C/T)(A/G)(C/T)(A/T)GTG on (-) strand] is shown in red and bold. Mismatches from consensus are underlined. Single nucleotide mutations are indicated in grey. The nucleotides inserted between the proximal BTG1 and the distal breakpoints are indicated. (PDF) [file pgen.1002533.s004.pdf]

Table S2. *BTG1* deletion breakpoint sequences in BTG1 MLPA deletion-positive BCP-ALL cases and cell lines.

Patient/Cell line	Sequence	Additional Nucleotides	Deletion
**Proximal (exon 2 *BTG1*)**			
Reference	TCAGGAGCTGTTCAGGCTTCTCCCAAGTGAACTCACACTCTGGG		
BCP-ALL 1008	TCAGGAGCTGTTCAGGCTTCTCCCAAGTGA	cccctccctgggggggcc	III
BCP-ALL 1014	TCAGGAGCTGTTCAGGCTTCTCCCAAGTGAACTCA	tag	III
BCP-ALL 1048	TCAGGAGCTGTTCAGGCTTCTCCCAAGTGAACTC	gg	III
BCP-ALL 1069	TCAGGAGCTGTTCAGGC	cggcccggaggg	III
BCP-ALL 1084	TCAGGAGCTGTTCAGGCTTCTCCCAAGTGAACTCA	tgggg	V
BCP-ALL 1086	TCAGGAGCTGTTCAGGCTTCTCCCAAGT	agaaggggacc	VI
BCP-ALL 1097	TCAGGAGCTGTTCAGGCTTCTCCCAAG	cccc	III
BCP-ALL 1134*1a	TCAGGAGCTGTTCAGGCTTCTCCCAAGTG	gaaa	III
BCP-ALL 1134*1b	TCAGGAGCTGTTCAGGCTTCTCCCAAGTGAACTCA	gg	III
BCP-ALL 1134*2	TCAGGAGCTGTTCAGGCTTCTCCCAAGTGAACT	ggggg	VIII
BCP-ALL 1135*1	TCAGGAGCTGTTCAGGCTTCTCCCAAGT	cctagg	I
BCP-ALL 1135*2	TCAGGAGCTGTTCAGGCTTCTCCCAAGTGAAC	a	III
BCP-ALL 1146*1a	TCAGGAGCTGTTCAGGCTTCTCCCAAGTGAA	g	III
BCP-ALL 1146*1b	TCAGGAGCTGTTCAGGCTTCTCCCAAGTGAACTCA	taa	III
BCP-ALL 1146*2	TCAGGAGCTGTTCAGGCTTCTCCC	ggggtta	V
BCP-ALL 1146*3	TCAGGAGCTGTTCAGGCTTCTCCCAAGTGAAC	cctc	VIII
BCP-ALL 1157*1	TCAGGAGCTGTTCAGGCTTCTCCCAAGTGA	tctct	III
BCP-ALL 1157*2	TCAGGAGCTGTTCAGGCTTCTCCCAAGTGAAC	ccccccaataggg	IV
BCP-ALL 1177	TCAGGAGCTGTTCAGGCTTC	cggg	III
BCP-ALL 1216	TCAGGAGCTGTTCAGGCTTCTCCCAAGT	cctcacgg	III
BCP-ALL 1238*1	TCAGGAGCTGTTCAGGCT	cct	III
BCP-ALL 1238*2	TCAGGA	cccttgaggg	V
BCP-ALL 1240*1	TCAGGAGCTGTTCAGGCTTC	gttcccc	II
BCP-ALL 1240*2	TCAGGAGCTGTTCAGGCTTCTCCCAAGTGAACTC	cgatttagga	III
BCP-ALL 1244*1	TCAGGAGCTGTTCAGGCTTCTCCCAAGTGAACT	ggg	III
BCP-ALL 1244*2	TCAGGAGCTGTTCAGGCTTCTCCCAAGTGAA	ccctg	V
BCP-ALL 1244*3	TCAGGAGCTGTTCAGGCTTCTCCCAAGTGAACTCA	gat	VIII
BCP-ALL 1264	TCAGGAGCTGTTCAGGCT	cggg	III
BCP-ALL 1273	TCAGGAGCTGTTCAGGCTTCTCCCAAGTGAACTCA	tgagca	III
BCP-ALL 1278	TCAGGAGCTGTTCAGGCTTCTCCCAAGTGAACT	tcatggggg	III
BCP-ALL 1315	TCAGGAGCTGTTCAGGCTTC	gttcccc	II
BCP-ALL 1316	TCAGGAGCTGTTCAGGCTTCTCCCAAGTGAACTCA	ttcg	V
BCP-ALL 1341*1	TCAGGAGCTGTTCAGGCTTC	gttcccc	II
BCP-ALL 1341*2	TCAGGAGCTGTTCAGGCTTCTCCCAAG	ccaacctcggggg	III
BCP-ALL 1341*3	TCAGGAGCTGTTCAGGCTTCTCCCAAG	taaaagt	V
BCP-ALL 1341*4	TCAGGAGCTGTTCAGGCTTCTCCCAAGTGAACT	ccttac	VIII
BCP-ALL 1344*1	TCAGGAGCTGTTCAGGCTTCTCCCAAGTG	gggga	III
BCP-ALL 1344*2a	TC	-	V
BCP-ALL 1344*2b	TCAGGAGCTGTTCAGGCTTCTCCCAAGTGAACTC	gag	V
BCP-ALL 1385	TCAGGAGCTGTTCAGG	ttcgcc	VIII
BCP-ALL 1411	TCAGGAGCTGTTCAGGCTTCTCCCAAGTGAACTCA	tggg	III
BCP-ALL 1442	TCAGGAGCTGTTCAGGCT	gcggggg	III
BCP-ALL 1457*1	TCAGGAGCTGTTCAGGCTTCTCCCAAGTGAACTC	cccttcccatttagatgaagg	III
BCP-ALL 1457*2	TCAGGAGCTGTTCAGGCTTCTCCCAAGTGAACTC	-	V
BCP-ALL 1471	TCAGGAGCTGTTCAGGCTTCTCCCAAGTGAACTC	-	V
BCP-ALL 1490	TCAGGAGCTGTTCAGGCTTC	gttcccc	II
BCP-ALL 1529	TCAGGAGCTGTTCAGGCTTCTCCCAAGTGAACTCA	tgg	III
BCP-ALL 1545*1	TCAGGAGCTGTTCAGGCTTCTCCCAAGTGAACTC	ccccccg	III

Patient/Cell line	Sequence	Additional Nucleotides	Deletion
**Proximal (exon 2 *BTG1*)**			
BCP-ALL 1545*2	TCAGGAGCTGTTCAGGCTTCTCCCAAGT	aag	V
BCP-ALL 1559*1	TCAGGAGCTGTTCAGGCTTCTCCCAAGTGAAC	ccctcgagag	III
BCP-ALL 1559*2	TCAGGAGCTGTTCAGGCTTCTCCCAAGTGAAC	cgcggg	VIII
BCP-ALL 1582	TCAGGAGCTGTTCAGGCTTCTCCCAAGTGAAC	cccaagg	III
BCP-ALL 1592	TCAGGAGCTGTTCAGGCTTCTCCCAAGTGAAC	ctgtt	III
BCP-ALL 1598	TCAGGAGCTGTTCAGGCTTCTCCCAAGTGAACTC	cgggggag	VII
BCP-ALL 1599	TCAGGAGCTGTTCAGGCTTCTCCCAAGTGA	cacttccc	III
BCP-ALL 1629*1	TCAGGAGCTGTTCAGGCTTCTCCCAAGTG	cccccct	III
BCP-ALL 1736*2	TCAGGAGCTGTTCAGGCTTCTCCCAAGTG	ggg	VIII
BCP-ALL 1630*1	TCAGGAGCTGTTCAGGCTTCTCCCAAGTGAACTCA	taggggggg	III
BCP-ALL 1630*2	TCAGGAGCTGTTCAGGCTTCTCCCAAGTG	gga	VIII
BCP-ALL 1654	TCAGGAGCTGTTCAGGCTTCTCCCAAGTGAACTC	cgggggag	VII
BCP-ALL 1736*1	TCAGGAGCTGTTCAGGCTTCTCCCAAGTGAACTCA	tccgg	III
BCP-ALL 1736*2	TCAGGAGCTGTTCAGGCTTCTCCCAAGTG	ggg	VIII
BCP-ALL 1749	TCAGGAGCTGTTCAGGCTTCTCCCAAGTGAA	aggggagggg	III
BCP-ALL 1755	TCAGGAGCTGTTCAGGCTTCTCCCAAGTGAACT	gg	VIII
BCP-ALL 1767	TCAGGAGCTGTTCAGGCTTC	gttcccc	II
BCP-ALL 1772*1	TCAGGAGCTGTTCAGGCTTC	aaggg	III
BCP-ALL 1772*2	TCAGGAGCTGTTCAGGCTTCTCCCAAGT	ccctcgt	VIII
BCP-ALL 1779	TCAGGAGCTGTTCAGGCTTCTCCCAAGTGAACTC	ttccc	V
BCP-ALL 1827	TCAGGAGCTGTTCAGGCTTC	gttcccc	II
BCP-ALL 1838	TCAGGAGCTGTTCAGGCTTCTCCCAAGTGAACTCA	tg	VIII
BCP-ALL 1853*1	TCAGGAGCTGTTCAGGCTTCTCCCAAGTGAACTCA	tgtt	III
BCP-ALL 1853*2	TCAGGAGCTGTTCAGGCTTCTCCC	ctc	IV
BCP-ALL 1865	TCAGGAGCTGTTCAGGCTTCTCC	ccccgc	III
BCP-ALL 1888	TCAGGAGCTGTTCAGGCTTCTCCCAAGTGAAC	gaag	III
BCP-ALL 1894	TCAGGAGCTGTTCAGGCTT	cccc	VIII
BCP-ALL 1896*1	TCAGGAGCTGTTCAGGCTTCTCCCAAGTGAACTC	tt	III
BCP-ALL 1896*2	TCAGGAGCTGTTCAGGCTTCTCCCAAGTGAACTCA	ga	VIII
Cell line 380	TCAGGAGCTGTTCAGGCTTC	gttccc	II
Cell line Mutz5	TCAGGAGCTGTTCAGGCTTCTCCCAAGTGAACT	ag	IV
Cell line REH	TCAGGAGCTGTTCAGGCTTCTCCCAAGTGAAC	cccgg	III
Cell line SupB15	TCAGGAGCTGTTCAGGCTTCTCCCAAGTGAACTCA	cca	IV
**Distal deletion I**			
Reference	GAGCTATGC**TACTGTG**TAGCTTAGGACAACTCGTTTGTCTGCTCTGGTACTCAAGT		
BCP-ALL 1135*1	GCTTAGGACAACTCGTTTGTCTGCTCTGGTACTCAAGT		
**Distal deletion II**			
Reference	CATACCTGTGTACGTGTTTAATTTCCTTTTTTTTTTTTTTTTTTTTTTGAGACAGA		
Cell line 380	TTTTTTTTTTTTTTTTTGAGACAGA		
BCP-ALL 1240*1	TTTTTTTTTTTTTTTTTGAGACAGA		
BCP-ALL 1315	TTTTTTTTTTTTGAGACAGA		
BCP-ALL 1341*1	TTTTTTTTTTTTTATGAGACAGA		
BCP-ALL 1490	TTTTTTTTTTTTTTTTGAGACAGA		
BCP-ALL 1767	TTTTTTTTTTTTTTCTTGAGACAGA		
BCP-ALL 1827	TTTTTTTTTTTTTTTTTTGAGACAGA		
**Distal deletion III (most frequent recombination hotspot)**			
Reference	AAGATGTTC**CATTGTG**TGAAACAGCGGCTCCTAGCTTTCATACATTTCCAGGATCA		
BCP-ALL 1008	CGGCTCCTAGCTTTCATACATTTCCAGGATCA		
BCP-ALL 1014	ACAGCGGCTCCTAGCTTTCATACATTTCCAGGATCA		
BCP-ALL 1048	GAAACAGCGGCTCCTAGCTTTCATACATTTCCAGGATCA		
BCP-ALL 1069	CAGCGGCTCCTAGCTTTCATACATTTCCAGGATCA		
BCP-ALL 1097	GAAACAGCGGCTCCTAGCTTTCATACATTTCCAGGATCA		

Patient/Cell line	Sequence	Additional Nucleotides	Deletion
**Distal deletion III (most frequent recombination hotspot)**			
BCP-ALL 1134*1a	GCGGCTCCTAGCTTTCATACATTTCCAGGATCA		
BCP-ALL 1134*1b	CGGCTCCTAGCTTTCATACATTTCCAGGATCA		
BCP-ALL 1135*2	AGCGGCTCCTAGCTTTCATACATTTCCAGGATCA		
BCP-ALL 1146*1a	GCGGCTCCTAGCTTTCATACATTTCCAGGATCA		
BCP-ALL 1146*1b	GCGGCTCCTAGCTTTCATACATTTCCAGGATCA		
BCP-ALL 1157*1	AGCGGCTCCTAGCTTTCATACATTTCCAGGATCA		
BCP-ALL 1177	GGCTCCTAGCTTTCATACATTTCCAGGATCA		
BCP-ALL 1216	TAGCTTTCATACATTTCCAGGATCA		
BCP-ALL 1238*1	AACAGCGGCTCCTAGCTTTCATACATTTCCAGGATCA		
BCP-ALL 1240*2	TGAAACAGCGGCTCCTAGCTTTCATACATTTCCAGGATCA		
BCP-ALL 1244*1	GCGGCTCCTAGCTTTCATACATTTCCAGGATCA		
BCP-ALL 1264	ACAGCGGCTCCTAGCTTTCATACATTTCCAGGATCA		
BCP-ALL 1273	GCGGCTCCTAGCTTTCATACATTTCCAGGATCA		
BCP-ALL 1278	GCGGCTCCTAGCTTTCATACATTTCCAGGATCA		
BCP-ALL 1341*2	ACAGCGGCTCCTAGCTTTCATACATTTCCAGGATCA		
BCP-ALL 1344*1	TGAAACAGCGGCTCCTAGCTTTCATACATTTCCAGGATCA		
BCP-ALL 1411	AACAGCGGCTCCTAGCTTTCATACATTTCCAGGATCA		
BCP-ALL 1442	GCGGCTCCTAGCTTTCATACATTTCCAGGATCA		
BCP-ALL 1457*1	GCGGCTCCTAGCTTTCATACATTTCCAGGATCA		
BCP-ALL 1529	AACAGCGGCTCCTAGCTTTCATACATTTCCAGGATCA		
BCP-ALL 1545*1	GCGGCTCCTAGCTTTCATACATTTCCAGGATCA		
BCP-ALL 1559*1	AGCGGCTCCTAGCTTTCATACATTTCCAGGATCA		
BCP-ALL 1582	GCGGCTCCTAGCTTTCATACATTTCCAGGATCA		
BCP-ALL 1592	AAACAGCGGCTCCTAGCTTTCATACATTTCCAGGATCA		
BCP-ALL 1599	CAGCGGCTCCTAGCTTTCATACATTTCCAGGATCA		
BCP-ALL 1629	AAACAGCGGCTCCTAGCTTTCATACATTTCCAGGATCA		
BCP-ALL 1630*1	CAGCGGCTCCTAGCTTTCATACATTTCCAGGATCA		
BCP-ALL 1736*1	GAAACAGCGGCTCCTAGCTTTCATACATTTCCAGGATCA		
BCP-ALL 1749	AACAGCGGCTCCTAGCTTTCATACATTTCCAGGATCA		
BCP-ALL 1772*1	GAAACAGCGGCTCCTAGCTTTCATACATTTCCAGGATCA		
BCP-ALL 1853*1	ACAGCGGCTCCTAGCTTTCATACATTTCCAGGATCA		
BCP-ALL 1865	GCGGCTCCTAGCTTTCATACATTTCCAGGATCA		
BCP-ALL 1888	CAGCGGCTCCTAGCTTTCATACATTTCCAGGATCA		
BCP-ALL 1896*1	CAGCGGCTCCTAGCTTTCATACATTTCCAGGATCA		
Cell line REH	GAAATAGCGGCTCCTAGCTTTCATACATTTCCAGGATCA		
**Distal deletion IV**			
Reference	GAAGAAAAC**AGCTGTG**TGCAGATAGACAATAGGGAAAAGGCACAGGAAGAGGAACT		
Cell line Mutz5	GATAGACAATAGGGAAAAGGCACAGGAAGAGGAACT		
Cell line SupB15	TGCAGATAGACAATAGGGAAAAGGCACAGGAAGAGGAACT		
BCP-ALL 1157*2	CAATAGGGAAAAGGCACAGGAAGAGGAACT		
BCP-ALL 1853*2	GATAGACAATAGGGAAAAGGCACAGGAAGAGGAACT		
**Distal deletion V (third frequent recombination hotspot)**			
Reference	CCAATGCAC**TGCAGTG**CCAGCTCCAGGGCCAGGCAGTAAATATTTGTTGAATGAAA		
BCP-ALL 1084	CCAGGGCCAGGCAGTAAATATTTGTTGAATGAAA		
BCP-ALL 1146*2	CCAGGGCCAGGCAGTAAATATTTGTTGAATGAAA		
BCP-ALL 1238*2	AGGGCCAGGCAGTAAATATTTGTTGAATGAAA		
BCP-ALL 1244*2	CAGCTCCAGGGCCAGGCAGTAAATATTTGTTGAATGAAA		
BCP-ALL 1316	GCTCCAGGGCCAGGCAGTAAATATTTGTTGAATGAAA		
BCP-ALL 1341*2	CCAGGGCCAGGCAGTAAATATTTGTTGAATGAAA		
BCP-ALL 1344*2a	CCAGGGCCAGGCAGTAAATATTTGTTGAATGAAA		
BCP-ALL 1344*2b	CCAGCTCCAGGGCCAGGCAGTAAATATTTGTTGAATGAAA		
BCP-ALL 1457*2	CAGCTCCAGGGCCAGGCAGTAAATATTTGTTGAATGAAA		

Patient/Cell line	Sequence	Additional Nucleotides	Deletion
**Distal deletion V (third frequent recombination hotspot)**			
BCP-ALL 1471	GCTCCAGGGCCAGGCAGTAAATATTTGTTGAATGAAA		
BCP-ALL 1545*2	GGGGCAGGCAGTAAATATTTGTTGAATGAAA		
BCP-ALL 1779	CCAGCTCCAGGGCCAGGCAGTAAATATTTGTTGAATGAAA		
**Distal deletion VI**			
Reference	GGGAATGGC**CTCTGTG**CCTGTCAGCTTGGACTTTCCAAAGTCAGCAGGCTGGAATG		
BCP-ALL 1086	GTCAGCTTGGACTTTCCAAAGTCAGCAGGCTGGAATG		
**Distal deletion VII**			
Reference	AGATAAATT**CACAGTG**TAAAAGCAATTATGATTTCCATAGATATTGCCAATGTCAA		
BCP-ALL 1598	ATGATTTCCATAGATATTGCCAATGTCAA		
BCP-ALL 1654	ATGATTTCCATAGATATTGCCAATGTCAA		
**Distal deletion VIII (second frequent recombination hotspot)**			
Reference	TAACGTTAT**CACTGTG**GGACAGCGTAGTGTGTTTGGGAAAACTTAGTCTTTTTTGT		
BCP-ALL 1134*2	AGCGTAGTGTGTTTGGGAAAACTTAGTCTTTTTTGT		
BCP-ALL 1146*3	GACAGCGTAGTGTGTTTGGGAAAACTTAGTCTTTTTTGT		
BCP-ALL 1244*3	AGCGTAGTGTGTTTGGGAAAACTTAGTCTTTTTTGT		
BCP-ALL 1341*4	GCGTAGTGTGTTTGGGAAAACTTAGTCTTTTTTGT		
BCP-ALL 1385	GACAGCGTAGTGTGTTTGGGAAAACTTAGTCTTTTTTGT		
BCP-ALL 1559*2	AGCGTAGTGTGTTTGGGAAAACTTAGTCTTTTTTGT		
BCP-ALL 1736*2	GCGTAGTGTGTTTGGGAAAACTTAGTCTTTTTTGT		
BCP-ALL 1630*2	AGCGTAGTGTGTTTGGGAAAACTTAGTCTTTTTTGT		
BCP-ALL 1736*2	GCGTAGTGTGTTTGGGAAAACTTAGTCTTTTTTGT		
BCP-ALL 1755	CAGCGTAGTGTGTTTGGGAAAACTTAGTCTTTTTTGT		
BCP-ALL 1772*2	ACAGCGTAGTGTGTTTGGGAAAACTTAGTCTTTTTTGT		
BCP-ALL 1838	CAGCGTAGTGTGTTTGGGAAAACTTAGTCTTTTTTGT		
BCP-ALL 1894	GGACAGCGTAGTGTGTTTGGGAAAACTTAGTCTTTTTTGT		
BCP-ALL 1896*2	ACAGCGTAGTGTGTTTGGGAAAACTTAGTCTTTTTTGT		

Sequencing of intragenic *BTG1* deletions demonstrates the presence of (near) consensus DNA sequence motifs for V(D)J recombination flanking the breakpoint hotspot in exon 2 of *BTG1* and the distal breakpoint clusters. The consensus heptamer RSS [CAC(A/T)(A/G)(C/T)(A/G) on (+) strand and (C/T)(A/G)(C/T)(A/T)GTG on (-) strand] is shown in red and bold. Mismatches from consensus are underlined. Single nucleotide mutations are indicated in grey. The nucleotides inserted between the proximal *BTG1* and the distal breakpoints are indicated.
