# Supplementary material for: The Origin and Nature of Tightly Clustered BTG1 Deletions in Precursor B-Cell Acute Lymphoblastic Leukemia Support a Model of Multiclonal Evolution
Source: PLoS Genet. 2012 Feb 16;8(2):e1002533. doi: 10.1371/journal.pgen.1002533 (PMC3280973; doi:10.1371/journal.pgen.1002533)
Supplement: Table S3 — Number of unique BTG1 deletion-spanning sequences in the BTG1 MLPA deletion-positive BCP-ALL cases and cell lines. (PDF) [file pgen.1002533.s005.pdf]

Table S3. Number of unique *BTG1* deletion-spanning sequences in the BTG1 MLPA deletion-positive BCP-ALL cases and cell lines.

Patient/Cell line	Deletion								Total
	I	II	III	IV	V	VI	VII	VIII	
BCP-ALL 1008	0	0	1	0	0	0	0	0	1
BCP-ALL 1014	0	0	1	0	0	0	0	0	1
BCP-ALL 1036	0	0	0	0	0	0	0	0	0
BCP-ALL 1048	0	0	1	0	0	0	0	0	1
BCP-ALL 1069	0	0	1	0	0	0	0	0	1
BCP-ALL 1084	0	0	0	0	1	0	0	0	1
BCP-ALL 1086	0	0	0	0	0	1	0	0	1
BCP-ALL 1093	0	0	0	0	0	0	0	0	0
BCP-ALL 1097	0	0	1	0	0	0	0	0	1
BCP-ALL 1134	0	0	2	0	0	0	0	1	3
BCP-ALL 1135	1	0	1	0	0	0	0	0	2
BCP-ALL 1146	0	0	2	0	1	0	0	1	4
BCP-ALL 1157	0	0	1	1	0	0	0	0	2
BCP-ALL 1176	0	0	0	0	0	0	0	0	0
BCP-ALL 1177	0	0	1	0	0	0	0	0	1
BCP-ALL 1198	0	0	0	0	0	0	0	0	0
BCP-ALL 1216	0	0	1	0	0	0	0	0	1
BCP-ALL 1238	0	0	1	0	1	0	0	0	2
BCP-ALL 1240	0	1	1	0	0	0	0	0	2
BCP-ALL 1244	0	0	1	0	1	0	0	1	3
BCP-ALL 1264	0	0	1	0	0	0	0	0	1
BCP-ALL 1272	0	0	0	0	0	0	0	0	0
BCP-ALL 1273	0	0	1	0	0	0	0	0	1
BCP-ALL 1274	0	0	0	0	0	0	0	0	0
BCP-ALL 1278	0	0	1	0	0	0	0	0	1
BCP-ALL 1304	0	0	0	0	0	0	0	0	0
BCP-ALL 1315	0	1	0	0	0	0	0	0	1
BCP-ALL 1316	0	0	0	0	1	0	0	0	1
BCP-ALL 1341	0	1	1	0	1	0	0	1	4
BCP-ALL 1344	0	0	1	0	2	0	0	0	3
BCP-ALL 1385	0	0	0	0	0	0	0	1	1
BCP-ALL 1411	0	0	1	0	0	0	0	0	1
BCP-ALL 1439	0	0	0	0	0	0	0	0	0
BCP-ALL 1442	0	0	1	0	0	0	0	0	1
BCP-ALL 1446	0	0	0	0	0	0	0	0	0
BCP-ALL 1454	0	0	0	0	0	0	0	0	0
BCP-ALL 1457	0	0	1	0	1	0	0	0	2
BCP-ALL 1471	0	0	0	0	1	0	0	0	1
BCP-ALL 1490	0	1	0	0	0	0	0	0	1
BCP-ALL 1499	0	0	0	0	0	0	0	0	0
BCP-ALL 1529	0	0	1	0	0	0	0	0	1
BCP-ALL 1545	0	0	1	0	1	0	0	0	2
BCP-ALL 1559	0	0	1	0	0	0	0	1	2
BCP-ALL 1582	0	0	1	0	0	0	0	0	1
BCP-ALL 1592	0	0	1	0	0	0	0	0	1
BCP-ALL 1598	0	0	0	0	0	0	1	0	1
BCP-ALL 1599	0	0	1	0	0	0	0	0	1
BCP-ALL 1629	0	0	1	0	0	0	0	1	2

Patient/Cell line	Deletion								Total
	I	II	III	IV	V	VI	VII	VIII	
BCP-ALL 1630	0	0	1	0	0	0	0	1	2
BCP-ALL 1654	0	0	0	0	0	0	1	0	1
BCP-ALL 1714	0	0	0	0	0	0	0	0	0
BCP-ALL 1736	0	0	1	0	0	0	0	1	2
BCP-ALL 1749	0	0	1	0	0	0	0	0	1
BCP-ALL 1755	0	0	0	0	0	0	0	1	1
BCP-ALL 1767	0	1	0	0	0	0	0	0	1
BCP-ALL 1772	0	0	1	0	0	0	0	1	2
BCP-ALL 1779	0	0	0	0	1	0	0	0	1
BCP-ALL 1827	0	1	0	0	0	0	0	0	1
BCP-ALL 1838	0	0	0	0	0	0	0	1	1
BCP-ALL 1845	0	0	0	0	0	0	0	0	0
BCP-ALL 1853	0	0	1	1	0	0	0	0	2
BCP-ALL 1865	0	0	1	0	0	0	0	0	1
BCP-ALL 1888	0	0	1	0	0	0	0	0	1
BCP-ALL 1894	0	0	0	0	0	0	0	1	1
BCP-ALL 1896	0	1	1	0	0	0	0	1	3
Cell line 380	0	1	0	0	0	0	0	0	1
Cell line Mutz5	0	0	0	1	0	0	0	0	1
Cell line REH	0	0	1	0	0	0	0	0	1
Cell line SupB15	0	0	0	1	0	0	0	0	1
Total	1	8	40	4	12	1	2	14	82
