# Supplementary material for: The Origin and Nature of Tightly Clustered BTG1 Deletions in Precursor B-Cell Acute Lymphoblastic Leukemia Support a Model of Multiclonal Evolution
Source: PLoS Genet. 2012 Feb 16;8(2):e1002533. doi: 10.1371/journal.pgen.1002533 (PMC3280973; doi:10.1371/journal.pgen.1002533)
Supplement: Table S4 — Subclonal BTG1 microdeletion occurrence within the cytogenetic subgroups. All patients screened were BTG1 deletion-negative as determined by MLPA. Subclonal BTG1 deletion status was determined using the PCR-based detection of deletions III, V and VIII. aBecause of missing values, numbers do not always add up to 89 BCP-ALL cases. Data was available for 77 cases on hyperdiploidy; 41 cases for ETV6-RUNX1; 60 cases for BCR-ABL1; 74 cases for MLL-rearrangement. bThe other subgroup encompasses cases negative for ETV6-RUNX1, MLL, BCR-ABL1 translocations and/or hyperdiploidy. This group does not contain any E2A-PBX1 translocation cases. cSubgroup unknown includes all cases in which no data is available in one or more cytogenetic subgroups. dFisher's exact test was used when sample groups were small. (PDF) [file pgen.1002533.s006.pdf]

Table S4. Subclonal *BTG1* microdeletion occurrence within the cytogenetic subgroups.

	Number of Samples[a]	*BTG1* deletion in minor subclone (%)	No *BTG1* deletion (%)	P-value Chi-square
T-ALL	77	0 (0)	77 (100)	<0.001
BCP-ALL	89	16 (18.0)	73 (82.0)	
Cytogenetic subgroup				0.064
Hyperdiploid (>50chr)				0.030
Yes	19	0 (0)	19 (100)	
No	58	13 (22.4)	45 (77.6)	
ETV6-RUNX1				0.033
Yes	8	4 (50.0)	4 (50.0)	
No	33	5 (15.2)	28 (84.8)	
BCR-ABL1				1.000[d]
Yes	1	0 (0)	1 (100)	
No	59	10 (16.9)	49 (83.1)	
MLL rearranged				1.000[d]
Yes	1	0 (0)	1 (100)	
No	73	11 (15.1)	62 (84.9)	
Other subgroup[b]				0.452
Yes	23	5 (21.7)	18 (78.3)	
No	29	4 (13.8)	25 (86.2)	
Subgroup unknown[c]				0.845
Yes	37	7 (18.9)	30 (81.1)	
No	52	9 (17.3)	43 (82.7)	

All patients screened were *BTG1* deletion-negative as determined by MLPA. Subclonal *BTG1* deletion status was determined using the PCR-based detection of deletions III, V and VIII. [a]Because of missing values, numbers do not always add up to 89 BCP-ALL cases. Data was available for 77 cases on hyperdiploidy; 41 cases for *ETV6-RUNX1*; 60 cases for *BCR-ABL1;* 74 cases for *MLL*-rearrangement. [b]The other subgroup encompasses cases negative for *ETV6-RUNX1*, *MLL*, *BCR-ABL1* translocations and/or hyperdiploidy. This group does not contain any *E2A-PBX1* translocation cases. [c]Subgroup unknown includes all cases in which no data is available in one or more cytogenetic subgroups. [d]Fisher's exact test was used when sample groups were small.
