# Supplementary material for: The Origin and Nature of Tightly Clustered BTG1 Deletions in Precursor B-Cell Acute Lymphoblastic Leukemia Support a Model of Multiclonal Evolution
Source: PLoS Genet. 2012 Feb 16;8(2):e1002533. doi: 10.1371/journal.pgen.1002533 (PMC3280973; doi:10.1371/journal.pgen.1002533)
Supplement: Table S5 — BTG1 fusion transcript sequences detected in independent subclones. Deletion spanning sequence was confirmed by sequencing of genomic DNA of the same case. (PDF) [file pgen.1002533.s007.pdf]

**Table S5.** *BTG1* fusion transcript sequences detected in independent subclones.

| Patient | BTG1 Sequence | Additional Nucleotides | Distal Sequence |
|---|---|---|---|
| **Deletion III** | | | |
| Pt1 (BCP-ALL 1888)[a] | GAGCTGTTCAGGCTTCTCCCAAGTGAAC | gaag | CAGCGGCTCCTAGCTTTCATACATTTCCAGG |
| Pt2 (BCP-ALL 1918) | GAGCTGTTCAGGCTTCTCCCA | gcatgagg | ACAGCGGCTCCTAGCTTTCATACATTTCCAGG |
| Pt3 (BCP-ALL 1959)[a] | GAGCTGTTCAGGCTTCTCCCAAGTGA | ccgg | AGCGGCTCCTAGCTTTCATACATTTCCAGG |
| Pt4 (BCP-ALL 1958)[a] | GAGCTGTTCAGGCTTCTCCCAAG | a | GGCTCCTAGCTTTCATACATTTCCAGG |
| Pt5 (BCP-ALL 1982)[a] | GAGCTGTTCAGGCTTCTCCCAAGTGAAC | gc | AACAGCGGCTCCTAGCTTTCATACATTTCCAGG |
| Pt6 (BCP-ALL 1457)[a] | GAGCTGTTCAGGCTTCTCCCAAGTGAACTC | cccttcccatttagatgaagg | AACAGCGGCTCCTAGCTTTCATACATTTCCAGG |
| **Deletion V** | | | |
| Pt1 (BCP-ALL 1888) | GAGCTGTTCAGGCTTCTCCCAAGTGA | ccacc | CCAGCTCCAGGGCCAGGCAGTAAATATTTGTTGA |
| Pt2 (BCP-ALL 1918) | GAGCTGTTCAGGCTTCTCC | ggaac | GGGCCAGGCAGTAAATATTTGTTGA |
| Pt4 (BCP-ALL 1958) | GAGCTGTTCAGGCTTCTCCCAAGTGAACTC | tccgtcagg | CCAGCTCCAGGGCCAGGCAGTAAATATTTGTTGA |
| Pt6 (BCP-ALL 1457)[a] | GAGCTGTTCAGGCTTCTCCCAAGTGAACTC | - | CAGCTCCAGGGCCAGGCAGTAAATATTTGTTGA |
| **Deletion VIII** | | | |
| Pt2 (BCP-ALL 1918) | GAGCTGTTCAGGCTTCTCCCAA | cccccg | GACAGCGTAGTGTGTTTGGGAAAACTTAGTCTTTT |
| Pt4 (BCP-ALL 1958) | GAGCTGTTCAGGCTTCTCCCA | tccag | GACAGCGTAGTGTGTTTGGGAAAACTTAGTCTTTT |

[a]Deletion spanning sequence was confirmed by sequencing of genomic DNA of the same case.
