# Supplementary material for: The Origin and Nature of Tightly Clustered BTG1 Deletions in Precursor B-Cell Acute Lymphoblastic Leukemia Support a Model of Multiclonal Evolution
Source: PLoS Genet. 2012 Feb 16;8(2):e1002533. doi: 10.1371/journal.pgen.1002533 (PMC3280973; doi:10.1371/journal.pgen.1002533)
Supplement: Table S6 — MLPA probes for copy-number analysis of the BTG1 gene region. Universal M13 PCR primers are indicated in bold. (PDF) [file pgen.1002533.s008.pdf]

Table S6. MLPA probes for copy-number analysis of the *BTG1* gene region.

Name	Location	Sequence*
BTG1_A	CLLUO1	5′- **GGGTTCCCTAAGGGTTGGA**CAAATTGGGGCACAACGAACTTAAGGAATGCCTTAAGACTGCC-3′
		5′-ACTGGTAAGTAACAGGGGTTAATTAGAACCCCACAGTGAGGGT**TCTAGATTGGATCTTGCTGGCAC**-3′
BTG1_B	*BTG1* before ex1	5′-**GGGTTCCCTAAGGGTTGGA**TCCAGCTACGCCACTTCCTTTTC-3′
		5′- GTGGCACTATAAAGGGTGCTGCA**TCTAGATTGGATCTTGCTGGCAC-3′**
BTG1_C	*BTG1* ex2	5′-**GGGTTCCCTAAGGGTTGGA**CACTCTGGGTTGACCCCTATGAAGT-3′
		5′-GTCCTACAGAATTGGAGAGGATGGC**TCTAGATTGGATCTTGCTGGCAC-3′**
BTG1_D	*BTG1* ex2	5′-**GGGTTCCCTAAGGGTTGGA**AGACACTGTCTCAACTGTGGTGTTAGCAC-3′
		5′-CAGCCAGCTCTCTGTACATTTGCTAGCTT**TCTAGATTGGATCTTGCTGGCAC-3′**
BTG1_E	*BTG1* 3′	5′-**GGGTTCCCTAAGGGTTGGA** TGTCATCTCCCACTAGTTTCTAGCACACGGGCT-3′
		5′-ACACACTCACTGGCCCACTTACCCTCTATTCTG**TCTAGATTGGATCTTGCTGGCAC-3′**
BTG1_F	*BTG1* 3′	5′-**GGGTTCCCTAAGGGTTGGA** GAGTGCTTCCTCTGATTACTCTGGATTTGTTGGTG-3′
		5′-CGTAATTTTAAAGTGGGGCTCTGTGGTTTCCCCAT**TCTAGATTGGATCTTGCTGGCAC-3′**
BTG1_G	*BTG1* 3′	5′-**GGGTTCCCTAAGGGTTGGA** CGGAAAGATATAGTGGAGGGAAGATGTCCATGGCAGGGATG-3′
		5′-TTGGAGGCACTGTGTCCAAACACGTTGGTAGGCTAGTACTG**TCTAGATTGGATCTTGCTGGCAC-3′**
BTG1_H	*BTG1* 3′	5′-**GGGTTCCCTAAGGGTTGGA** CACCATGGACAGCAGGGCTGTATTTATTACC-3′
		5′-ATCCTAATGGCAAGTCATTCAGTGAGCCGCA**TCTAGATTGGATCTTGCTGGCAC-3′**
BTG1_I	DCNEX1	5′- **GGGTTCCCTAAGGGTTGGA**CTGTCAATGCCATCTTCGAGTGGTCCAGTGTTCTGATTT-3′
		5′- GGGTGAGTGGGATGCAGTTTCTCTTTACCTACCTTACTT**TCTAGATTGGATCTTGCTGGCAC**-3′

* Universal M13 PCR primers are indicated in bold
