# Supplementary material for: The Origin and Nature of Tightly Clustered BTG1 Deletions in Precursor B-Cell Acute Lymphoblastic Leukemia Support a Model of Multiclonal Evolution
Source: PLoS Genet. 2012 Feb 16;8(2):e1002533. doi: 10.1371/journal.pgen.1002533 (PMC3280973; doi:10.1371/journal.pgen.1002533)
Supplement: Table S7 — Primers used for BTG1 bisulfite sequencing, mutation screening, breakpoint mapping, expression analysis and CHIP assay. (PDF) [file pgen.1002533.s009.pdf]

Table S7. Primers used for *BTG1* bisulfite sequencing, mutation screening, breakpoint mapping, expression analysis and CHIP assay.

	Forward primer	Reverse primer
**Bisulfite sequencing**		
	5’-TTGTTTTGGAGTGTAAGTGTTGG-3’	5’-TACAATCCTTTTTCTTAAACAAAAC-3’
***BTG1* sequencing**		
Exon 1	5’-GACTCTGACCCAGGGATGTG-3’	5’-CATCACGCTCCAGCTACG-3’
Exon 2	5’-TCCATAATCCATCCCCAAGA-3’	5’-GGATGCAATCCTGGACATTT-3’
***BTG1* breakpoint mapping**		
Deletion I	5’-TAGGATCTGCCCCAATGTTC-3’	5’-GGATCCTCTGATTGGACAGG-3’
Deletion II	5’-TGTACACATAAATGCTGATGAACTC-3’	5’-TCACTGGTTCCCAGAAAAGC-3’
Deletion III	5’-CATGACATTTTAGGAGGACGTT-3’	5’-TCATTTTCGCTCCTCAAAGG-3’
Deletion IV	5’-ACACTCTACTTTCATACTTTGTAA-3’	5’-TCACTGGTTCCCAGAAAAGC-3’
Deletion V	5’-TCACTGAAGTGTGGCAATGG-3’	5’-TCATTTTCGCTCCTCAAAGG-3’
Deletion VI	5’-CCCAGATCCATTCCTTCTCA-3’	5’-TCACTGGTTCCCAGAAAAGC-3’
Deletion VII	5’-GGCTGCATTTTTGTTGATGA-3’	5’-TCACTGGTTCCCAGAAAAGC-3’
Deletion VIII	5’-CACTTCCCTTCACCCATTGT-3’	5’-TCATTTTCGCTCCTCAAAGG-3’
**RT-PCR**		
WT-BTG1-Fwd A	5'-ATGCATCCCTTCTACACCCGG-3'	
WT-BTG1-Rev B		5’-AATTCTGTAGGACACTTCATAGGG-3’
Del II BTG1^{trunc} –Rev C		5’-TTGCAGCGAGCTGGGATTGCGCCA-3’
Del III BTG1^{trunc} –Rev D		5’-TTGATCCTGGAAATGTATGAAAGC-3’
Del IV BTG1^{trunc} –Rev E		5’-TGGATAAAAGAAGATGAGACCAGA-3’
Del VIII BTG1^{trunc} –Rev F		5’-CCAAAGCAGCCTCATTGCCTAATG-3’
Del VIII BTG1^{trunc} –Rev G		5’-ACCCTCAACATAGATCATCAGTTA-3’
**Real-time qRT-PCR primers**		
BTG1ex1/2	5’-TGACGAGCGAGCGACAGCTGCA-3’	5’-GCATGGCTTTTCTGGGAACCAGTG-3’
BTG1ex2	5’-AGCACAGCGGATTGGACTGAGCAG-3’	5’-AATTCTGTAGGACACTTCATAGGG-3’
HPRT	5’- TGACACTGGCAAAACAATGCA-3’	5’- GGTCCTTTTCACCAGCAAGCT-3’
**Cloning and expression *BTG1* construct**		
HA-BTG1-Fwd	5’-GCGAATTCGACCATGGCTTACCCATACGATGTTCCAGATTACGCTCATCCCTTCTACACCCGGG-3’	
BTG1-Trunc-Rev	5’-GCCTCGAGTCATTAACTTGGGAGAAGCCTGAACA-3’	
TBP control	5’- GCACAGGAGCCAAGAGTGAA-3’	5’- ACATCACAGCTCCCCACCAT-3’
**ChIP primers**		
-1kb prom	5’-CGCCTTCCTGTTACACTGGTTTTT-3’	5’-CAAATGCCGTCCTAGCCTGAGTCC-3’
Prox prom	5’-AACCGGCTTGCGTCACAATGGTGC-3’	5’-GCAGCACCCTTTATAGTGCCACGA-3’
Exon 2	5’-TATTCGCATCAACCATAAAATGGA-3’	5’-AATTCTGTAGGACACTTCATAGGG-3’
3’ÚTR	5’-AGATAGTTTGTAATACCAGCCGAC-3’	5’-AAGATCTATAGGCATAACCAGAAA-3’
